# Amorphous/Crystalline Heterostructured Nanomaterials: An Emerging Platform for Electrochemical Energy Storage

**DOI:** 10.1002/smll.202411941

**Published:** 2025-02-28

**Authors:** Yan Zhou, Yihua Liang, Zhen Wu, Xinlei Wang, Runnan Guan, Changqing Li, Fen Qiao, Junfeng Wang, Yongsheng Fu, Jong‐Beom Baek

**Affiliations:** ^1^ School of Energy and Power Engineering Jiangsu University Zhenjiang 212013 China; ^2^ School of Energy and Chemical Engineering/Center for Dimension Controllable Organic Frameworks Ulsan National Institute of Science and Technology (UNIST) 50 UNIST Ulsan 44919 South Korea; ^3^ School of Energy and Power Engineering Chongqing University Chongqing 400044 China; ^4^ Key Laboratory for Soft Chemistry and Functional Materials of Ministry of Education Nanjing University of Science and Technology Nanjing 210094 China

**Keywords:** amorphous/crystalline heterostructure, electrochemical energy storage, interface effects, synergistic interactions

## Abstract

With the expanding adoption of large‐scale energy storage systems and electrical devices, batteries and supercapacitors are encountering growing demands and challenges related to their energy storage capability. Amorphous/crystalline heterostructured nanomaterials (AC‐HNMs) have emerged as promising electrode materials to address these needs. AC‐HNMs leverage synergistic interactions between their amorphous and crystalline phases, along with abundant interface effects, which enhance capacity output and accelerate mass and charge transfer dynamics in electrochemical energy storage (EES) devices. Motivated by these elements, this review provides a comprehensive overview of synthesis strategies and advanced EES applications explored in current research on AC‐HNMs. It begins with a summary of various synthesis strategies of AC‐HNMs. Diverse EES devices of AC‐HNMs, such as metal‐ion batteries, metal–air batteries, lithium–sulfur batteries, and supercapacitors, are thoroughly elucidated, with particular focus on the underlying structure–activity relationship among amorphous/crystalline heterostructure, electrochemical performance, and mechanism. Finally, challenges and perspectives for AC‐HNMs are proposed to offer insights that may guide their continued development and optimization.

## Graphical abstract

This is a comprehensive review of amorphous/crystalline heterostructured nanomaterials (AC‐HNMs), highlighting their synthesis strategies and applications in electrochemical energy storage devices, including metal‐ion batteries, metal–air batteries, lithium–sulfur batteries, and supercapacitors. The structure–activity relationships among amorphous/crystalline heterostructure, performance, and mechanism are thoroughly analyzed. Challenges and future perspectives for the optimization of AC‐HNMs are also proposed.

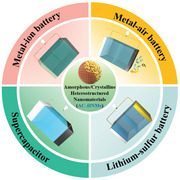

## 1 Introduction

Vigorous efforts to develop renewable energy have become a major strategic component in the global energy transition away from fossil fuels and as a response to climate change.^[^ ^1^, ^2^, ^3^ ^]^ However, to provide a stable energy supply most renewable energy sources (like solar, wind, and tidal power) also require the use of advanced electrical energy storage systems because of factors such as geographical constraints and intermittency.^[^ ^4^, ^5^ ^]^ Therefore, to realize the efficient use of these clean energy sources, the development of low‐cost, high‐energy, high‐power electrochemical energy storage (EES) technologies has become particularly important. In this regard, steady advances in EES applications have been made in recent decades, including the development of metal‐ion batteries,^[^ ^6^, ^7^, ^8^, ^9^ ^]^ metal–air batteries,^[^ ^10^, ^11^, ^12^, ^13^ ^]^ lithium–sulfur batteries,^[^ ^14^, ^15^, ^16^ ^]^ and supercapacitors.^[^ ^17^, ^18^, ^19^ ^]^ Despite these great achievements, it remains challenging for present EES devices to meet the growing demand for practical applications. One of the main bottlenecks that prevents these EES devices from being more widely adopted is the poor activity of their electrode materials. To this end, the exploration of highly efficient electrode materials is urgently needed.

To meet the requirements of various EES systems, precise regulation of the compositions, dimensionality, morphology, architecture, and sizes of electrode materials is necessary.^[^ ^20^, ^21^, ^22^, ^23^, ^24^, ^25^ ^]^ Phase control provides an effective and powerful method of constructing highly efficient electrode materials because it allows for distinct atomic arrangements as well as the modification of electronic structure, compositions, dimensionality, morphology, architecture, and sizes.^[^ ^26^, ^27^, ^28^ ^]^ Crystalline nanomaterials, which generally feature an ordered and periodic arrangement of atoms, possess abundant reaction interfaces and active facets because of their directional arrangement and closely packed structure.^[^ ^29^, ^30^, ^31^ ^]^ Amorphous nanomaterials, characterized by a disordered arrangement of atoms, display percolation pathways, isotropic ion diffusion channels, and unsaturated coordination environment, which offers fast ion diffusion and high active site density.^[^ ^32^, ^33^ ^]^ Nevertheless, it is important to note that pure‐phase nanomaterials have their limitations. The ion diffusion pathways in crystalline nanomaterials are relatively fixed, hindering rapid ion migration and leading to lower kinetic performance.^[^ ^34^, ^35^ ^]^ Furthermore, their typically limited number of active sites constrains electrochemical performance and overall reactivity.^[^ ^11^, ^36^ ^]^

Compared to crystalline nanomaterials, the relatively lower electrical conductivity of amorphous nanomaterials typically results in an additional energy barrier and corresponding poor kinetics, posing an obstacle to their further advancement.^[^ ^37^ ^]^

**Figure 1:**
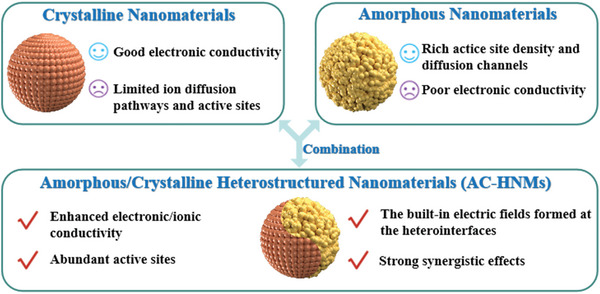
Comparison of the properties of crystalline nanomaterials, amorphous nanomaterials, and AC‐HNMs.

As a result, efforts to effectively overcome the limitations inherent in single amorphous or crystalline nanomaterials have recently focused on the creation of amorphous/crystalline heterostructured nanomaterials (AC‐HNMs) given their potential (**Figure** **1**). The synergistic interactions between the different phases provide greater opportunities to tune the properties of the materials. On the one hand, the amorphous component can furnish abundant active sites and ion diffusion channels, thereby enhancing the electrochemical activity of the electrode materials.^[^ ^38^, ^39^ ^]^ On the other hand, the crystalline part, characterized by high electronic conductivity, ensures swift charge transfer, consequently improving the electrical conductivity of the electrode materials.^[^ ^40^, ^41^ ^]^ In particular, the highly lattice‐mismatched configuration of the amorphous and crystalline phases generates numerous dangling bonds, distorted atomic structures, and tunable electronic structures.^[^ ^42^ ^]^ These highly unsaturated atomic bonds and differences in chemical potential between the different components help drive the electrons near the interface to move directionally until the Fermi energy levels on both sides are equal.^[^ ^36^ ^]^ The simultaneous presence of electron‐rich and positively charged regions leads to a charge distribution gradient and the formation of a built‐in electric field.^[^ ^43^ ^]^ The built‐in electric field formed at the amorphous/crystalline heterointerface lowers the reaction energy barriers, provides additional active storage sites, and effectively regulates the charge transfer kinetics.^[^ ^37^, ^44^ ^]^

**Figure 2:**
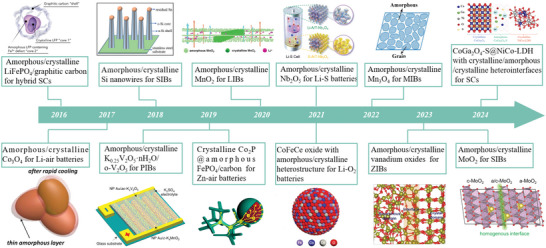
Schedule of representative results for AC‐HNMs in energy storage devices from 2016 to 2024.^[^ ^45^, ^46^, ^47^, ^48^, ^49^, ^50^, ^51^, ^52^, ^53^, ^54^, ^55^, ^56^ ^]^ Copyright 2016, Wiley‐VCH; Copyright 2017, Wiley‐VCH; Copyright 2018, The Royal Society of Chemistry; Copyright 2019, Springer Nature; Copyright 2019, American Chemical Society; Copyright 2020, Wiley‐VCH; Copyright 2021, The Royal Society of Chemistry; Copyright 2021, Wiley‐VCH; Copyright 2022, Elsevier; Copyright 2016, Wiley‐VCH; Copyright 2023, Elsevier; Copyright 2024, American Chemical Society.

With continuous effort, by designing novel electrode materials with amorphous/crystalline heterostructures that match the target EES devices, researchers have successfully constructed high‐performance metal‐ion batteries, metal–air batteries, lithium–sulfur batteries, and supercapacitors (**Figure** **2**).^[^ ^45^, ^46^, ^47^, ^48^, ^49^, ^50^, ^51^, ^52^, ^53^, ^54^, ^55^, ^56^ ^]^ However, while considerable research on energy‐related applications of AC‐HNMs has been reported, there is still a lack of in‐depth, focused analysis of their specific applications to EES. In addition, unlike previous reviews that either cover a wide range of applications or focus on electrocatalytic reactions, this review is exclusively dedicated to AC‐HNMs for EES applications, to provide a more comprehensive and detailed introduction and discussion.

**Figure 3:**
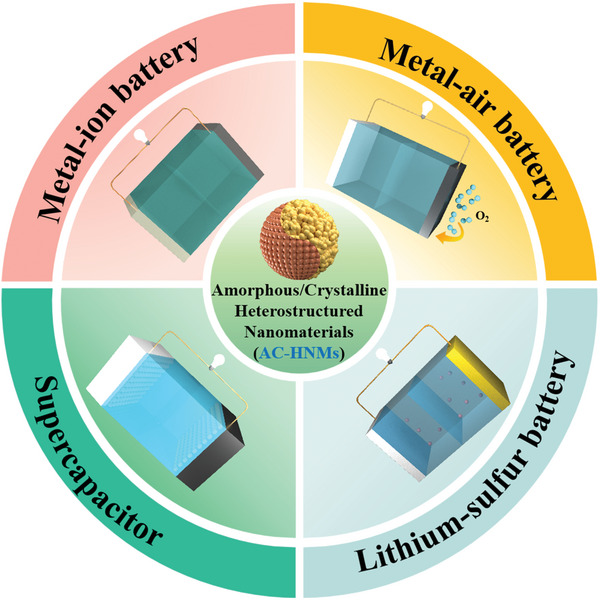
Schematic illustration of AC‐HNMs for various EES applications.

Here, we provide the latest advances in the rapidly evolving landscape of AC‐HNMs, with particular interest in synthetic methods, and especially EES applications. In the beginning, we discuss synthesis methods from the perspective of how the crystalline and amorphous phases in AC‐HNMs are formed. Following that, we comprehensively emphasize the utilization of AC‐HNMs as highly efficient electrodes in various EES applications, including metal‐ion batteries, metal–air batteries, lithium–sulfur batteries, and supercapacitors (**Figure** **3**), while also clarifying the structure–activity relationships among amorphous/crystalline heterostructure, performance, and mechanism. Finally, we discuss current challenges and perspectives on the future research and development of AC‐HNMs for EES applications. We anticipate that this timely review will assist researchers in the development of more optimal AC‐HNMs and in gaining a deeper understanding of the structure–activity relationships among amorphous/crystalline heterostructure, performance, and mechanism.

## 2 Synthesis Methods of AC‐HNMs

The synthesis of AC‐HNMs is considered challenging because of the thermodynamically unstable characteristic of long‐range disordered atomic structures in amorphous compositions.^[^ ^37^, ^57^ ^]^ Many synthesis methods have been explored for the construction of AC‐HNMs, including the wet‐chemical method,^[^ ^58^, ^59^, ^60^, ^61^ ^]^ thermal treatment method,^[^ ^62^, ^63^, ^64^ ^]^ and electrochemical method,^[^ ^65^, ^66^, ^67^, ^68^ ^]^ and more. In this section, we aim to provide a concise summary to assist researchers in selecting appropriate methods for creating AC‐HNMs. Given the presence of both crystalline and amorphous phases in AC‐HNMs, without considering the synthesis of the precursor materials, the reported methods can be categorized into two groups based on the formation mode of the crystalline and amorphous phases: one is the one‐step method, and the other is the stepwise method.

### 2.1 The One‐Step Method

The one‐step methods involve the simultaneous formation of both crystalline and amorphous phases in the synthesis of AC‐HNMs, without considering the synthesis step of the precursor. Undoubtedly, utilizing a one‐step process for the synthesis of AC‐HNMs can greatly reduce time expenditure, making it more appealing for practical applications. The critical factor in achieving a one‐step synthesis of AC‐HNMs is the precise control of experimental parameters, including reactants, temperature, pressure, and solvents. By optimizing the reaction kinetic parameters, it becomes possible to simultaneously generate both crystalline and amorphous regions during the synthesis process.

**Figure 4:**
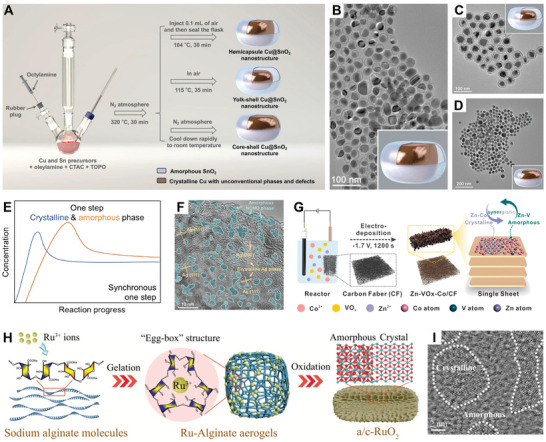
A) Schematic illustration of the synthesis of amorphous SnO_2_‐encapsulated crystalline Cu heterostructures. TEM images of B) hemicapsule Cu@SnO_2_, C) yolk–shell Cu@SnO_2_, and D) core–shell Cu@SnO_2_ nanostructures. (B–D) Inset: schematic illustration of three types of heterostructures. Reproduced with permission.^[^ ^60^ ^]^ Copyright 2022, Wiley‐VCH. E) Schematic of synchronous synthesis process for amorphous/crystalline heterostructure nanomaterials. F) HRTEM image of crystalline Ag/amorphous NiCoMo oxides. Reproduced with permission.^[^ ^71^ ^]^ Copyright 2021, Wiley‐VCH. G) Schematic illustration of the preparation of Zn–VO*_x_*–Co 2D ultrathin nanosheets. Reproduced with permission.^[^ ^73^ ^]^ Copyright 2022, Elsevier B.V. H) Synthesis procedure and I) atomic‐resolution HAADF‐STEM image of a/c‐RuO_2_. Reproduced with permission.^[^ ^74^ ^]^ Copyright 2021, Wiley‐VCH.

In 2022, Zhang's group reported the synthesis of three distinct amorphous SnO_2_‐encapsulated crystalline Cu heterostructures (hemicapsule Cu@SnO_2_, yolk–shell Cu@SnO_2_, and core–shell Cu@SnO_2_ nanostructures) using a straightforward one‐pot wet‐chemical method (**Figure** **4**A–D).^[^ ^60^ ^]^ The treatment of the solution under different experimental conditions was the key to obtaining three types of amorphous/crystalline heterostructures. In another study, Wang's team developed 0D/2D amorphous/crystalline heterostructures, consisting of amorphous BiO*_x_* nanodots embedded within metallic bismuth nanoflakes (BiO*_x_*/Bi‐nf), using a simple one‐step reduction method.^[^ ^69^ ^]^ Specifically, by optimizing the NaBH_4_ content, Bi^3+^ ions were reduced to create 2D metallic bismuth nanoflakes (Bi‐nf), while partially reduced amorphous BiO*_x_* nanodots became incorporated within the Bi‐nf, resulting in the desired BiO*_x_*/Bi‐nf with amorphous/crystalline heterostructures. Similarly, a one‐step reduction method was also adopted by Deng et al. to synthesize a RuB aerogel with amorphous/crystalline heterostructures, which was achieved through the in situ reduction of RuCl_3_ using NaBH_4_.^[^ ^70^ ^]^ The crystalline phase structure of the resulting RuB aerogel could be effectively modulated by controlling the concentration of NaBH_4_.

In hydrothermal and solvothermal methods, elevated pressure and temperature conditions enable the simultaneous formation of amorphous and crystalline phases due to their distinct reaction kinetics.^[^ ^37^ ^]^ For instance, Li et al. reported a one‐pot hydrothermal reaction in which crystalline Ag and amorphous NiCoMo oxides coprecipitate to create crystalline Ag/amorphous NiCoMo oxides with dense crystalline–amorphous interfacial sites (Figure 4E,F).^[^ ^71^ ^]^ The elevated temperature and pressure conditions in the hydrothermal method facilitate the combination of the formation of two phases with distinct reaction kinetics into a one‐step process. Similarly, Wang and colleagues employed a one‐pot hydrothermal method to synthesize amorphous Mo‐doped NiS_0.5_Se_0.5_@crystalline NiS_0.5_Se_0.5_ nanorods on nickel foam.^[^ ^72^ ^]^ During this process, the synergistic effects of hydrazine hydrate and sodium borohydride as reducing agents led to the formation of amorphous/crystalline heterostructures. Besides water‐based thermal reactions, organic solvent‐based thermal reactions, known as solvothermal reactions, can be used to construct AC‐HNMs. Sun and colleagues synthesized amorphous/crystalline CoFeCe oxide in ethanol through a straightforward one‐step solvothermal reaction, without the necessity for subsequent annealing.^[^ ^52^ ^]^ The precise control of the Co, Fe, and Ce content was the crucial factor in achieving the synthesis of amorphous/crystalline CoFeCe oxides.

In addition to hydrothermal/solvothermal methods, electrodeposition offers an alternative approach to one‐pot fabrication of AC‐HNMs, thanks to its controllable reaction kinetics. Chen and co‐workers employed a facile electrodeposition method to prepare 2D nanosheets composed of amorphous VO*_x_*–Co and crystalline Zn–Co phases with a well‐integrated heterostructure (Figure 4G).^[^ ^73^ ^]^ During the electrodeposition process, the inclusion of doped VO*_x_* clusters led to a highly disordered lattice in the metallic Co, while the doping of Zn prevented the amorphization of the Co species. Simultaneously, the doping of both Zn and VO*_x_* generated a suitable amorphous/crystalline heterostructure with abundant interfaces between the crystalline ZnCo phases and amorphous VO*_x_*–Co.

The thermal annealing strategy, another effective one‐step synthesis technique, also enables the production of AC‐HNMs. Zhang and co‐workers prepared sodium‐decorated amorphous/crystalline RuO_2_ with rich oxygen vacancies (a/c‐RuO_2_) from Ru‐alginate hydrogel using a thermal oxidation treatment at 450 °C for 3 h (Figure 4H).^[^ ^74^ ^]^ Atomic‐resolution high‐angle annular dark‐field scanning transmission electron microscopy (HR‐HAADF‐STEM) image revealed the existence of obvious amorphous/crystalline heterointerfaces (Figure 4I). It should be noted that an appropriate thermal oxidation time and temperature are required to form a/c‐RuO_2_. Similarly, An et al. fabricated amorphous NiO/crystalline NiCeO*_x_* by thermally annealing high‐Ni‐content binuclear coordination polymers of Ni and Ce in air.^[^ ^75^ ^]^ The coordination geometry of organic ligands was crucial to forming a high‐nickel‐content coordination polymer, enabling Ni supersaturation in the crystalline NiCeO*_x_* structure during annealing and facilitating the migration of Ni from the interior to the surface of the crystalline NiCeO*_x_* to form amorphous NiO. In another study, Tian et al. achieved the controlled conversion of Co(OH)_2_ into amorphous/crystalline CoO nanosheets using a vacuum‐calcination approach.^[^ ^76^ ^]^ The formation of amorphous/crystalline CoO originated from the dehydration of Co(OH)_2_. During the process, more oxygen vacancies were induced under reduced oxygen partial pressure in negative pressure conditions, resulting in the generation of amorphous phases due to incomplete phase transitions.

Chemical vapor deposition (CVD) represents yet another viable strategy for constructing amorphous/crystalline heterostructures. For instance, Yue et al. successfully synthesized Si_3_N_4_/SiO_2_ nanochain heterojunctions, comprising Si_3_N_4_ single crystal nanowires and amorphous SiO_2_ beads, using a one‐step CVD process.^[^ ^77^ ^]^ In this process, carbon vapor induced the formation of Si_3_N_4_ nanowires while inhibiting the formation of Si_2_N_2_O, and the synthesis of Si_3_N_4_/SiO_2_ nanochains was possible only within an optimal range of carbon vapor concentration.

Spraying techniques have been widely employed in the synthesis of AC‐HNMs. Wang and co‐workers synthesized crystalline Sn@amorphous SiOC core–shell nanoparticles (c‐Sn@a‐SiOC NPs) using a spray pyrolysis and one‐step carbonization method.^[^ ^78^ ^]^ Phase separation between Sn and diphenylsilanediol (DPSD) results in the formation of core@shell‐structured Sn@DPSD NPs, which are subsequently carbonized through heat treatment in an inert atmosphere to produce c‐Sn@a‐SiOC NPs. The amorphous SiOC coating effectively stabilized the crystalline Sn nanoparticles, maintaining their high reversible capacities while reducing the initial coulombic efficiency loss.

Mechanical ball milling provides a cost‐effective means of creating AC‐HNMs. Yu's group developed a series of tin (Sn)/phosphorus (P) matrix composites with amorphous/crystalline heterostructures using a simple and cost‐effective ball milling method.^[^ ^79^ ^]^ By changing the preparation parameters (the composition and proportion of the precursor material and rotation speeds), the transformation of Sn_4_P_3_ to crystalline Sn and amorphous P in the carbon skeleton could be well controlled to achieve the controllable amorphous/crystalline heterostructures. The dispersion of nanoscale Sn and/or Sn_4_P_3_ crystals within the amorphous P matrix formed a sultana pudding structure, which was favorable for synergistic sodium ion storage.

Despite the advantages of time economy and simplicity, the one‐step method is still limited by the inability to precisely control the amorphous/crystalline heterointerfaces, and its lack of scalability. Synthetic techniques that possess both controllable and scalable features are essential and must be developed for fundamental research and large‐scale applications in AC‐HNMs.

### 2.2 The Stepwise Method

Compared to the one‐step methods, the stepwise methods are more controlled and more common. Stepwise synthesis involves the sequential creation of amorphous and crystalline phases in AC‐HNMs. More specifically, in stepwise synthesis, either the crystalline phase is formed first, followed by the amorphous phase, or the amorphous phase is created initially, with the crystalline phase synthesized afterward. We categorize the stepwise methods reported in the literature into three distinct categories: secondary growth, partial amorphization of crystals, and partial crystallization of amorphous nanomaterials.

#### 2.2.1 Secondary Growth

**Figure 5:**
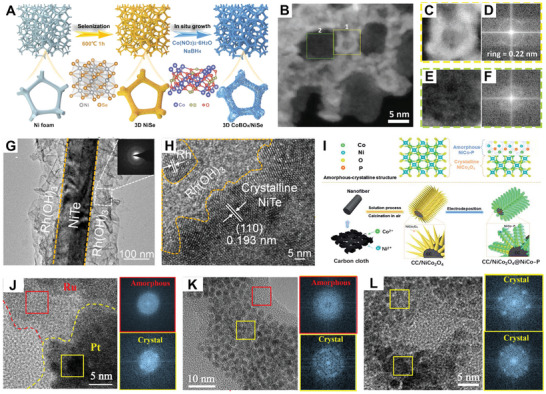
A) Schematic illustration of the synthesis process for 3D CoBO*_x_*/NiSe. Reproduced with permission.^[^ ^87^ ^]^ Copyright 2023, Wiley‐VCH. B) High‐magnification HAADF‐STEM images of Ni–B_i_/meso‐Ir heterostructures; C,D) Enlarged image and corresponding FFT from the area “1” of (B). E,F) Enlarged image and corresponding fast Fourier transform (FFT) from the area “2” of (B). Reproduced with permission.^[^ ^70^ ^]^ Copyright 2021, Wiley‐VCH. G) TEM images of a‐Rh(OH)_3_/NiTe, inset shows SAED patterns of amorphous Rh(OH)_3_. H) HRTEM images of a‐Rh(OH)_3_/NiTe. Reproduced with permission.^[^ ^89^ ^]^ Copyright 2022, Elsevier. I) Schematic illustration of the synthesis process of CC/NiCo_2_O_4_@NiCo‐P. Reproduced with permission.^[^ ^91^ ^]^ Copyright 2022, The Royal Society of Chemistry. TEM patterns and FFT patterns of J) 1cPt/aRu, K) 3cPt/aRu, and L) 5cPt/aRu. Reproduced with permission.^[^ ^93^ ^]^ Copyright 2023, American Chemical Society.

Secondary growth refers to the use of presynthesized nanomaterials as seeds, onto which new nanomaterials are deposited on their surfaces or interfaces. This method is particularly useful for synthesizing AC‐HNMs. One of them involves supporting the amorphous nanomaterials directly on pre‐synthesized crystalline nanomaterials to construct amorphous/crystalline heterostructures. Amorphous cobalt boride and nickel boride are commonly employed for hybridization with presynthesized crystalline nanomaterials.^[^ ^44^, ^80^, ^81^, ^82^ ^]^ This is because the introduction of boron (B) into non‐noble metals such as nickel (Ni) and cobalt (Co) during chemical reduction using strong reducing agents (such as sodium borohydride or dimethylamine borane) can effectively inhibit crystallization.^[^ ^83^, ^84^, ^85^, ^86^, ^87^ ^]^ For example, Liu and co‐workers created a 3D self‐standing CoBO*_x_*/NiSe with amorphous/crystalline heterostructures using a two‐step procedure (**Figure** **5**A).^[^ ^87^ ^]^ First, nickel foam was selenidized to generate the crystalline NiSe. Subsequently, amorphous CoBO*_x_* nanosheets were grown on the 3D NiSe using a wet‐chemical reduction strategy, resulting in the final amorphous/crystalline heterostructures. In another study, Asahi's group designed AC‐HNMs consisting of crystalline mesoporous iridium (meso‐Ir) and amorphous nickel–boron oxide (Ni–B_i_) using a two‐step wet‐chemical reduction strategy.^[^ ^84^ ^]^ Specifically, the meso‐Ir nanosheets were first synthesized using polystyrene–polyethylene oxide block copolymer micelles as templates and formic acid as a reducing agent. Then Ni–B_i_ was coated with low‐temperature sodium borohydride solution as a reducing agent on the surfaces of the prepared meso‐Ir nanosheets to form Ni–B_i_/meso‐Ir with amorphous/crystalline heterostructures. As observed in Figure 5B, most of the Ir nanocrystalline domains (bright contrasts) are present on the pore walls of the nanosheets, while darker contrasts indicated a thin layer covering both the Ir matrix and the pores. The enlarged images in Figure 5C,E further revealed the crystalline structure of the Ir skeleton, revealing a lattice spacing of 0.22 nm that corresponds to the (111) plane of the face‐centered cubic (fcc) structures (Figure 5D). Meanwhile, the Ni–B_i_ layer covering the pores was identified as amorphous (Figure 5F). Similarly, Karthik and co‐workers initially synthesized square rod‐shaped crystalline manganese molybdate (MnMoO_4_) through coprecipitation and heat treatment. The MnMoO_4_/Ni*_x_* B heterostructures were then created by embedding amorphous nickel boride (Ni*_x_* B) onto the surface of MnMoO_4_ rods through a chemical reduction method.^[^ ^88^ ^]^

Beyond well‐known amorphous nanomaterials like cobalt boride and nickel boride, other amorphous nanomaterials can also be anchored to presynthesized crystalline nanomaterials under specific conditions to create AC‐HNMs. For instance, Sun et al. developed a novel core–shell nanorod array composed of crystalline NiTe and amorphous Rh(OH)_3_ using a hydrothermal method followed by a chemical etching process.^[^ ^89^ ^]^ In this approach, NiTe nanoarrays were initially grown on nickel foam through hydrothermal synthesis, where the NF served as both the substrate and nickel source, while N_2_H_4_ acted as a reducing agent, facilitating the reaction of Na_2_TeO_3_ and nickel to form crystalline NiTe nanorods. Following this, the NiTe nanorods were subjected to chemical etching in an aqueous RhCl_3_ *x*H_2_O solution. This process primarily targeted the surface of the crystalline NiTe nanorods, with Cl^−^ ions accelerated etching and induced the in situ formation of an amorphous Rh(OH)_3_ layer on the NiTe surfaces. Figure 5G showed a distinct crystalline nanorod enveloped by an amorphous sheath, featuring a crystalline NiTe core and an amorphous Rh(OH)_3_ shell. The amorphous/crystalline heterointerfaces could also be clearly observed in Figure 5H. Similarly, an amorphous/crystalline Rh(OH)_3_/CoP heterostructure was constructed by Xing and co‐workers.^[^ ^90^ ^]^ The results demonstrated that the successful construction of the amorphous/crystalline heterointerfaces imparted Rh(OH)_3_/CoP with unique hydrophilic/aerophobic properties, optimal defect structures, excellent conductivity, and strong corrosion resistance.

In another case, amorphous NiCo‐(HPO_4_)_2_ H_2_O (NiCo‐P) ultrathin nanosheets were in situ grown on crystalline NiCo_2_O_4_ nanowires by Gao and co‐workers (Figure 5I).^[^ ^91^ ^]^ First, crystalline NiCo_2_O_4_ was directly grown on carbon cloth via a hydrothermal method with a further calcination process. Then NiCo‐P ultrathin nanosheets were uniformly anchored on the NiCo_2_O_4_ nanowires via an in situ electrodeposition technique, forming CC/NiCo_2_O_4_@NiCo‐P with distinctive amorphous/crystalline heterostructures. Similarly, Fan et al. also constructed a 0D/2D amorphous/crystalline heterostructure, where amorphous iron‐based spinel oxide (a‐MFe_2_O_4_, with M = Ni, Co, Zn) was uniformly anchored on the crystalline exfoliated black phosphorus (c‐EBP) nanosheets using electrochemical and solvothermal methods.^[^ ^92^ ^]^ Specifically, bulk black phosphorus was first exfoliated into c‐EBP through electrochemical exfoliation, followed by the uniform immobilization of a‐MFe_2_O_4_ onto the c‐EBP via a solvothermal process, resulting in the a‐MFe_2_O_4_@c‐EBP with amorphous/crystalline heterostructures.

Another approach involves directly supporting crystalline nanomaterials on presynthesized amorphous nanomaterials to create amorphous/crystalline heterostructures. For instance, Lou and collaborators synthesized bimetal PtRu nanoparticles with crystalline Pt modification on amorphous RuO*_x_* (c‐Pt/a‐Ru NPs).^[^ ^93^ ^]^ As shown in Figure 5J–L, increasing the Pt content within the c‐Pt/a‐Ru altered the coverage pattern of crystalline Pt from island‐like to cross‐linkable, and eventually to dense coverage. This progression enhanced the exposure of amorphous/crystalline heterointerfaces in the 3c‐Pt/a‐Ru configuration. Similarly, Wang et al. prepared Pd/a‐MnO_2_ with abundant amorphous/crystalline heterointerfaces using a rapid two‐step wet chemical method.^[^ ^94^ ^]^ In this process, amorphous MnO_2_ nanosheets (a‐MnO_2_) were synthesized by reducing KMnO_4_ with NaH_2_PO_2_, and the subsequent addition of H_2_PdCl_4_ led to the formation of ultrafine crystalline palladium (Pd) nanoparticles on the a‐MnO_2_ surfaces. The a‐MnO_2_, rich in surface defects, provided key nucleation sites for Pd, facilitating the growth of Pd nanocrystals. In a separate study, Zhu et al. first developed an amorphous CoMoS*_x_* hollow nanotube array on carbon cloth using a two‐step hydrothermal reaction. They then introduced crystalline Ru species onto the amorphous CoMoS*_x_* hollow nanotube array through Ru^3+^ ion etching followed by a subsequent annealing process, resulting in the formation of amorphous/crystalline heterostructures.^[^ ^95^ ^]^

#### 2.2.2 Partial Amorphization of Crystals

**Figure 6:**
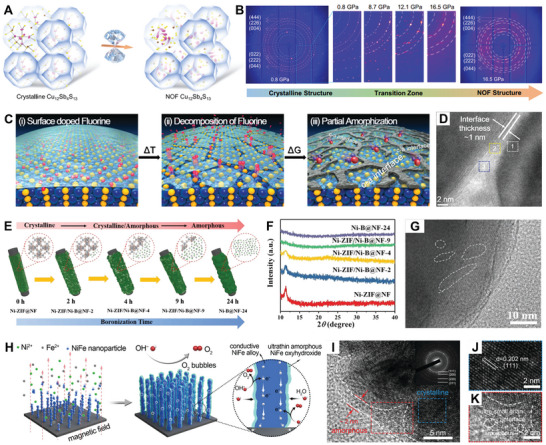
A) Pressure‐induced transformation of crystalline Cu_12_Sb_4_S_13_ to the nested order–disorder framework (NOF) structure. B) The single crystal XRD images. Reproduced with permission.^[^ ^96^ ^]^ Copyright 2022, Springer Nature. C) Schematic of the amorphous/crystalline interfaces via fluorine surface doping in cobalt boride. D) HRTEM image of the F‐Co_2_B. Reproduced with permission.^[^ ^97^ ^]^ Copyright 2019, The Royal Society of Chemistry. E) Schematic illustration of the synthetic process of Ni‐ZIF/Ni‐B@NF. F) XRD patterns of all the samples. G) HRTEM images of Ni‐ZIF/Ni‐B‐4. Reproduced with permission.^[^ ^100^ ^]^ Copyright 2019, Wiley‐VCH. H) Schematic of the synthesis of the Ni*_x_* Fe_1−_ *_x_*‐AHNA nanowire array. I–K) HRTEM images of Ni*_x_* Fe_1−_ *_x_*‐AHNA, the inset of (I) is the SAED pattern, the images (J,K) are enlarged views of the selected area in (I). Reproduced with permission.^[^ ^104^ ^]^ Copyright 2020, The Royal Society of Chemistry.

The partial amorphization of crystals refers to the partial transformation of a crystal into an amorphous structure by bombarding the crystal with a controlled external force (pressure, doping, etc.) that breaks part of the crystal lattice or induces defects. For example, Bu et al. achieved a framework structure characterized by disordered–ordered nesting on the sublattice scale by the introduction of pressure to modulate the intrinsic chemical bonding levels in Cu_12_Sb_4_S_13_ crystals (**Figure** **6**A).^[^ ^96^ ^]^ As shown in Figure 6B, the increase in pressure led to a significant broadening of certain Bragg diffraction spots in the Cu_12_Sb_4_S_13_ crystals after reaching 12 GPa, signifying the initiation of disorder within a portion of the crystalline structure. By 16.5 GPa, this portion of the diffraction spot disappeared, becoming a diffuse diffraction band. Interestingly, some single‐crystal diffraction spots still remained on the amorphous diffraction ring, indicating that part of the crystalline structure was preserved, resulting in the formation of amorphous/crystalline heterostructures.

Doping is a common strategy to partially transform crystalline into amorphous nanomaterials. For example, Han et al. reported the synthesis of fluorine (F)‐doped cobalt boride (F‐Co_2_B) with high‐density amorphous/crystalline heterointerfaces through F doping (Figure 6C).^[^ ^97^ ^]^ As shown in Figure 6D,F was initially introduced into the surface sites of the prepared crystalline Co_2_B. Upon heating at elevated temperatures, the surface‐doped F underwent decomposition in the presence of air, resulting in the partial surface amorphization of Co_2_B, consequently leading to the creation of highly dense amorphous/crystalline heterointerfaces in the F‐Co_2_B. Similarly, Wang and co‐worker synthesized amorphous/crystalline S‐doped Pd nanosheet arrays on nickel foam by an ambient immersion strategy, to incorporate S doping into crystalline Pd nanosheet arrays.^[^ ^98^ ^]^ The amorphous/crystalline heterostructure might result from the rapid expansion of the Pd lattice due to the introduction of larger S atoms, which hindered the atomic arrangement of the Pd fcc structure, thereby partially transforming the crystalline region into an amorphous region. In another study, ultrathin oxygen‐doped FePSe_3_ (FePSe_3_‐O) nanosheets with abundant amorphous/crystalline heterointerfaces were prepared by Peng's group.^[^ ^99^ ^]^ Specifically, ultrathin crystalline FePSe_3_ nanosheets were first obtained through a chemical vapor transport method followed by ultrasonic exfoliation. Subsequently, the FePSe_3_‐O nanosheets were prepared by plasma treatment under Ar/O_2_. O atom doping was introduced into the nanosheets to induce the phase transformation, which was accompanied by the formation of locally disordered lattice structures.

Other techniques such as boronization, sulfurization, phosphorization, selenization, and oxidation can also induce partial amorphization in presynthesized crystalline nanomaterials. For instance, Xu et al. utilized a room‐temperature boronization method to partially convert crystalline nickel zeolite imidazolate (Ni‐ZIF) into Ni–B, creating amorphous/crystalline heterointerfaces (Figure 6E).^[^ ^100^ ^]^ They found that increasing the boronization time weakened the Ni‐ZIF diffraction peaks, eventually leading to a fully amorphous Ni–B phase (Figure 6F). Locally disordered lattice structures also emerged within the crystalline Ni‐ZIF phase after boronization with NaBH_4_ for 4 h, introducing numerous amorphous/crystalline heterointerfaces (Figure 6G). Using thermal phosphidation, Zhang and co‐workers created a layered amorphous/crystalline/amorphous structure.^[^ ^101^ ^]^ Specifically, crystalline NiMoO_4_ nanorods were first synthesized in situ on nickel foam via a hydrothermal method, and then an amorphous Ni_2_P/crystalline P‐doped NiMoO_4_/amorphous P‐doped NiMoO_4_ sandwich structure was constructed by controlling the reaction time and temperature during phosphidation. Similarly, Dong's group explored an in situ salinization strategy to create an ultrathin amorphous layer of Pt*_x_* Ru*_y_* Se*_z_* on the surface of ultrasmall PtRu nanocrystals.^[^ ^102^ ^]^ In this case, the inner core consisted of a Pt–Ru alloy crystal (c‐PtRu), while the outer layer represented an ultrathin amorphous layer (a‐Pt*_x_* Ru*_y_* Se*_z_*), which was similar to transplanting a layer of amorphous skin onto the surface of the alloy single crystal, showcasing the potential for enhanced interfacial properties. Recently, Wu et al. developed a crystalline Bi_2_Se_3_/amorphous BiO*_x_* heterostructure by oxidizing crystalline Bi_2_Se_3_ with H_2_O_2_.^[^ ^59^ ^]^ During this oxidation process, the surface of Bi_2_Se_3_ was transformed into amorphous BiO*_x_*, while the interior structure remained well‐preserved.

The electrochemical reconstruction method offers an effective approach for inducing partial amorphization in crystalline nanomaterials. In this technique, a thin surface layer of the crystalline nanomaterial is in situ transformed into an amorphous active phase at specific electrode potentials. For example, Liu and co‐workers fabricated an electrochemically activated cobalt nickel double hydroxide material (E‐CoNi DH) with abundant amorphous structure using an electrochemical method.^[^ ^103^ ^]^ Specifically, cobalt–nickel double hydroxide (CoNi DH) nanosheets were first electrodeposited on a 3D exfoliated graphite substrate. Then, the CoNi DH electrode was activated 200 times by cyclic voltammetry (CV) in a 0.5 m KOH electrolyte. After activation, H defects were induced in the hydroxide material, resulting in some amorphous domains in the E‐CoNi DH. In addition to Liu's work, Liang and co‐workers developed robust Ni*_x_* Fe_1−_ *_x_* alloy (core)‐ultrathin amorphous oxyhydroxide (shell) nanowire arrays via a magnetic‐field‐assisted chemical deposition method (Figure 6H).^[^ ^104^ ^]^ As shown in Figure 6I–K, an in situ formed amorphous NiFe oxyhydroxide layer, only 1–5 nm thick, coated the crystalline NiFe alloy nanowire surface. The amorphous NiFe hydroxide prepared by the electrochemical reconstruction method was much thinner than that prepared by electrodeposition, hydrothermal method, and chemical deposition method, which was beneficial to reducing the electron transfer resistance in the material and improving the charge transfer ability.

#### 2.2.3 Partial Crystallization of Amorphous Nanomaterials

**Figure 7:**
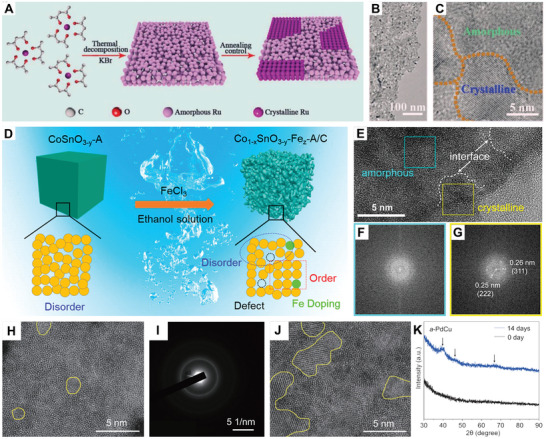
A) Schematic illustration of the synthesis of amorphous/crystalline Ru nanosheets. B) TEM image, and C) HRTEM image of amorphous/crystalline Ru nanosheets. Reproduced with permission.^[^ ^62^ ^]^ Copyright 2021, Wiley‐VCH. D) Schematic illustration of the synthesis of Co_1−_ *_x_* SnO_3−_ *_y_*‐Fe_z_‐A/C. E) HRTEM image of Co_1−_ *_x_* SnO_3−_ *_y_*‐Fe*_z_*‐A/C, and the corresponding FFT patterns of the selected regions marked by the F) blue and G) yellow squares, respectively. Reproduced with permission.^[^ ^106^ ^]^ Copyright 2023, American Chemical Society. H) The spherical aberration‐corrected HAADF‐STEM image and I) SAED pattern of the a‐PdCu nanosheets. J) The spherical aberration‐corrected HAADF‐STEM images of a‐PdCu after aging for 14 days. K) XRD patterns of a‐PdCu samples being aged for 0 day and 14 days. Reproduced with permission.^[^ ^107^ ^]^ Copyright 2019, Oxford University Press.

The partial crystallization of amorphous nanomaterials typically requires energy‐intensive methods and carefully controlled conditions, such as high‐temperature annealing and pressure. For example, Xu and Kong transformed amorphous ruthenium nanosheets into ruthenium nanosheets with amorphous/crystalline heterostructures through a controlled annealing method (**Figure** **7**A).^[^ ^62^ ^]^ In this process, the reaction temperature played an important role in regulating the crystallinity. Certain regions of the amorphous Ru nanosheets rapidly crystallized to achieve higher atomic order, while others remained amorphous, forming boundaries between different crystalline domains and leading to the attachment of small nanoflakes (Figure 7B,C). Similarly, Wu et al. employed a two‐step annealing method to synthesize 2D Fe_2_O_3_ nanosheets featuring amorphous/crystalline heterostructures.^[^ ^105^ ^]^ In the first step, amorphous Fe_2_O_3_ nanosheets were formed, followed by a second annealing at a moderate, controlled temperature to induce partial crystallization, resulting in a hybrid structure of amorphous and crystalline domains. This phase adjustment and material optimization were finely tuned by precise control of the thermal processing conditions.

In addition, researchers have explored several straightforward and eco‐friendly methods to induce partial amorphization in crystalline nanomaterials. For instance, Pan's group reported a facile strategy to tailor metal–oxygen bonds by rationally designing amorphous/crystalline heterostructures, which was achieved by subjecting amorphous CoSnO_3−_ *_y_* cubes to ion exchange in FeCl_3_ ethanol solution (Figure 7D).^[^ ^106^ ^]^ The ion‐exchange process strengthened the Sn─O bonds and weakened the Co─O bond strengths and generated additional Fe─O bonds, accompanied by abundant Co defects and appropriate oxygen defect concentrations, resulting in the formation of amorphous/crystalline heterostructures (Co_1−_ *_x_* SnO_3−_ *_y_*‐Fe*_z_*‐A/C, Figure 7E–G). In another study, Zhang's group reported an aging‐induced crystallization phenomenon in PdCu nanosheets with amorphous/crystalline heterostructures.^[^ ^107^ ^]^ In short, the amorphous phase‐dominant PdCu nanosheets (Figure 7H,I) were used as the starting material, synthesized via a one‐pot wet chemical method, and then aged in hexane under ambient conditions. After 14 days of aging, the crystallinity of the resulting amorphous/crystalline PdCu nanosheets increased significantly. This change was evidenced by the expansion of crystalline regions, as observed in the HAADF‐STEM image (Figure 7J) and the intensified XRD diffraction peaks (Figure 7K), indicating a transition from the amorphous to the crystalline state induced by aging.

## 3 Advanced Electrochemical Energy Storage Systems

AC‐HNMs, characterized by their enriched active sites, unsaturated coordination structures, fast ion diffusion channels, abundant phase boundaries, and formed built‐in electric fields at heterointerfaces, offer unique and advantageous properties as electrode materials in EES devices such as metal‐ion batteries, metal–air batteries, lithium–sulfur batteries, and supercapacitors. In the subsequent sections, we would like to summarize and discuss the rational design of the structure and chemical composition of AC‐HNMs and their applications in different types of EES devices.

### 3.1 Metal‐Ion Batteries

#### 3.1.1 Lithium‐Ion Batteries (LIBs)

Since their commercialization in 1991, LIBs have been widely used as essential energy storage systems in electric vehicles, portable electronic devices, and other fields.^[^ ^108^, ^109^, ^110^ ^]^ However, the current energy density of lithium‐ion batteries (LIBs) is insufficient to meet the requirements for grid‐scale electric applications.^[^ ^111^, ^112^ ^]^ This application for LIBs requires the development of advanced, high‐capacity electrode materials to replace the current options. Various advanced nanomaterials, particularly AC‐HNMs, have been employed as anodes for LIBs because of their distinct physicochemical properties.^[^ ^50^, ^113^, ^114^ ^]^ Their isotropic ion diffusion paths and the built‐in electric fields at heterointerfaces effectively enhance Li^+^ diffusion and electron transfer, while offering additional active sites, which are crucial for electrochemical reactions in LIBs.^[^ ^115^, ^116^ ^]^

**Figure 8:**
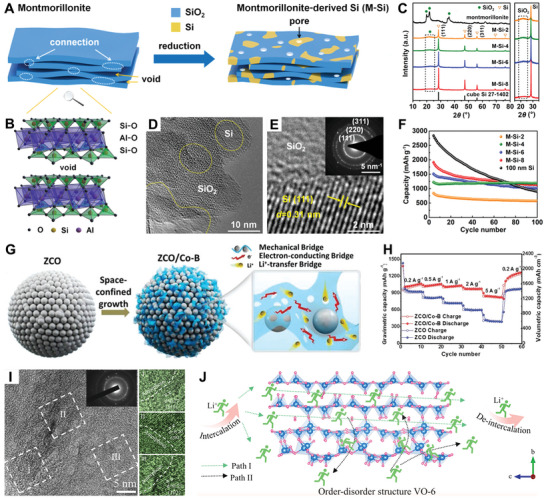
A) Schematic Illustration of montmorillonite montmorillonite‐derived Si (M‐Si). B) Crystal model of montmorillonite. C) XRD patterns of montmorillonite and M‐Si samples at different reduction times. D) TEM image of M‐Si‐4. E) HRTEM image with clear lattice fringes and SAED pattern (inset) of M‐Si‐4. F) Cycling stability of different Si samples at 0.2 C. Reproduced with permission.^[^ ^117^ ^]^ Copyright 2024, American Chemical Society. G) Schematic diagram of the multiple functions of Co–B nanoflakes in the ZCO/Co–B hybrid electrode. H) Rate performance of ZCO/Co–B and ZCO electrodes. Reproduced with permission.^[^ ^118^ ^]^ Copyright 2019, Wiley‐VCH. I) HRTEM image of order–disorder VO‐6 structure. J) The schematic diagram of the Li‐ion diffusion path of VO‐6. Reproduced with permission.^[^ ^115^ ^]^ Copyright 2024, Elsevier.

Very recently, Zhang et al. leveraged montmorillonite with staggered layered structures to produce a crystalline–amorphous Si/SiO_2_ anode via a controlled magnesiothermic reduction process (**Figure** **8**A,B).^[^ ^117^ ^]^ As the reduction time increased from 2 to 8 h, the characteristic Si peak intensities steadily increased, while the broad SiO_2_ peaks (≈22°) gradually weakened (Figure 8C), signifying a gradual transformation into Si domains. Under a reduction time of 4 h, the as‐prepared material (M‐Si‐4) exhibited the mixed phase of Si/SiO_2_, where the crystalline Si domains (10–30 nm) were embedded within the amorphous SiO_2_ network (Figure 8D,E). The crystalline Si regions offered Li storage capacity, while the amorphous SiO_2_ ensured local structural integrity and stability, forming a strong foundation for long‐term cycling performance. As illustrated in Figure 8F, the M‐Si‐4 anode demonstrated a relatively high charge capacity of ∼1200 mAh g^−1^, with almost no capacity decay after 100 cycles and a capacity retention of 97%. In another study, Wang and collaborators incorporated amorphous Co–B nanoflakes into crystalline ZnCo_2_O_4_ (ZCO) micro/nanospheres to form a tightly integrated ZCO/Co–B heterostructure for LIBs (Figure 8G).^[^ ^118^ ^]^ Electrochemical investigations have demonstrated that these ZCO/Co–B heterointerfaces not only enhance the mechanical durability and electronic conductivity of the electrode but also expedite Li^+^ diffusion, resulting in ZCO/Co–B anodes with superior lithium storage properties. As shown in Figure 8H, the ZnCo_2_O_4_/Co–B electrode delivered high gravimetric/volumetric capacities of 1051 mAh g^−1^/1576 mAh cm^−3^ at 0.2 A g^−1^, respectively, with robust rate capability.

Furthering this trend, Guo and co‐workers developed an ultrathin 2D leaf‐like structured amorphous/crystalline MnO_2_ as an anode material for LIBs.^[^ ^50^ ^]^ The 2D amorphous/crystalline heterostructure integrated flexible ultrathin amorphous sheets that enhanced ion transport, nonflexible crystalline skeletons that provided stable mechanical support and facilitated fast electronic transfer, and a porous framework that served as permeable channels to accelerate ion transport kinetics. This distinctive amorphous/crystalline leaf‐like design significantly improved the capacity and cycling lifespan of LIBs. In 2023, Wang and colleagues synthesized core–shell crystalline copper vanadates@amorphous copper vanadium oxide using a facile template strategy.^[^ ^119^ ^]^ When employed as an anode in LIBs, the prepared electrode demonstrated a substantial reversible capacity of 967 mAh g^−1^, outstanding rate performance (390 mAh g^−1^ at 5 A g^−1^), and remarkable cycling stability (98.2% retention after 200 cycles). This improved lithium storage was attributed to the synergistic effect of the amorphous copper vanadium oxide layer and the presence of abundant oxygen vacancies, which effectively mitigate volume expansion and promote pseudocapacitive behavior. Most recently, Cui and co‐workers designed an ordered–disordered (crystalline–amorphous) vanadium oxide anode for aqueous LIBs (ALIBs) using a calcination method.^[^ ^115^ ^]^ The as‐prepared ordered–disordered V_2_O_5_ (VO‐6) contains a significant number of both amorphous and crystalline regions (Figure 8I). This order–disorder structure of the VO‐6 provides high electronic conductivity and fast lithium‐ion diffusion, which synergistically accelerated electron/ion transport, reduced battery polarization, and lowered the reaction energy barrier with Li^+^. These effects enhanced redox reaction kinetics and ultimately improved electrochemical performance. As shown in Figure 8J, the ordered–disordered VO‐6, featuring 3D ion pathways, demonstrated rapid lithium ion diffusion capabilities, confirming that VO‐6 was one of the good candidate materials for developing fast‐charging anodes for ALIBs.

Moreover, Yang and co‐workers developed porous Si‐embedded amorphous carbon@graphitic carbon composite microspheres (Si/AC@GC) via a spray drying technique.^[^ ^120^ ^]^ The unique spherical structure, incorporating nanovoids to facilitate liquid electrolyte infiltration and a graphitic carbon layer for enhanced electrical conductivity, significantly enhances the Li^+^ storage capabilities of Si/AC@GC microspheres. The Si/AC@GC achieved a reversible capacity of 803 mAh g^−1^ at 1.0 A g^−1^ after 200 cycles, showcasing impressive cycling stability. Even at 5.0 A g^−1^, it maintained a reversible discharge capacity of 589 mAh g^−1^ with negligible capacity loss.

In addition, Esper et al. synthesized flake‐like glass (amorphous)–ceramic (crystalline) composites consisting of SiO_2_ and GeO_2_.^[^ ^121^ ^]^ Specifically, the flake‐shaped glass particles were formed through liquid‐phase compression via ball milling, followed by heat treatment to crystallize Ge*_x_* Si*_y_* O_6_ domains within the SiO_2_–GeO_2_ glass matrix. As an anode material for LIBs, the composites demonstrated a stable reversible capacity of 520 mAh g^−1^ at 0.2 C and maintained 87% of its capacity after 100 cycles. The excellent cycling stability resulted from the synergistic interaction between the nanocrystals, serving as active sites for electrochemical conversion and alloying reactions, and the glass matrix, which accommodates volume changes and alleviates stress during lithium insertion. Similarly, Li et al. developed a germanium phosphate glass containing precipitating GeO_2_ microcrystals for use as an anode material in LIBs.^[^ ^122^ ^]^ The presence of GeO_2_ crystals enhanced the specific surface area and offered additional reaction sites for lithiation reaction, thereby improving the anode's capacity. The anode achieved a discharge specific capacity of 740.9 mAh g^−1^ after 600 cycles at 500 mA g^−1^.

#### 3.1.2 Sodium‐Ion Batteries (SIBs)

With the explosive growth of the LIB market, the limited availability of lithium resources has gradually drawn attention.^[^ ^123^ ^]^ In contrast, sodium resources are abundant and low cost, making SIBs a highly promising alternative candidate in the energy storage field, potentially replacing LIBs in the future.^[^ ^9^, ^124^ ^]^ Regrettably, Na^+^ (0.102 nm) has a larger radius compared to Li^+^ (0.076 nm), leading to sluggish sodiation/desodiation kinetics and serious volume expansion.^[^ ^125^ ^]^ Therefore, for efficient sodium storage, it is essential to employ electrode materials that feature larger ion diffusion channels. AC‐HNMs are highly effective in SIBs, leveraging the amorphous phase's abundant active sites and Na⁺ transport channels, the crystalline phase's conductivity, and the built‐in electric fields to accelerate charge transport—all contributing to enhanced electrochemical performances.

**Figure 9:**
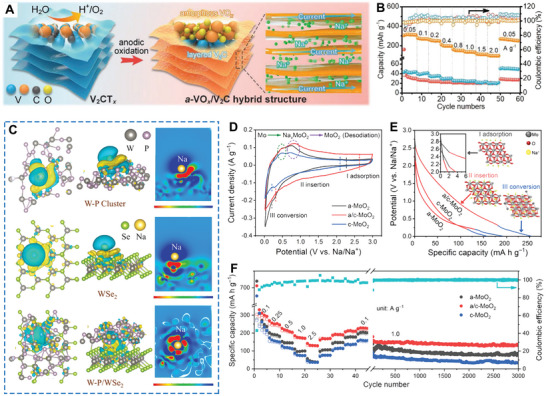
A) Schematic diagram illustrating the synthesis of a‐VO*_x_*/V_2_C heterostructures. B) Rate capability of a‐VO*_x_*/V_2_C. Reproduced with permission.^[^ ^126^ ^]^ Copyright 2021, Wiley‐VCH. C) Differential charge density distributions (various perspectives) and cross‐sectional contours of Na atoms adsorbed on model surfaces or interfaces in W–P clusters, WSe₂, and W–P/WSe₂. Reproduced with permission.^[^ ^127^ ^]^ Copyright 2024, Elsevier. D) Integrated CV curves at 0.25 mV s^−1^, E) reversible discharge profiles at 0.25 A g^−1^, and F) rate capability and cycling stability of the MoO_2_ electrodes. Reproduced with permission.^[^ ^55^ ^]^ Copyright 2023, Elsevier.

In 2021, Zhang and co‐workers constructed an amorphous vanadium oxide/V_2_C MXene (a‐VO*_x_*/V_2_C) heterostructure via the controllable constant voltage oxidation of multilayer V_2_CT*_x_* in an aqueous electrolyte (**Figure** **9**A).^[^ ^126^ ^]^ During the process, the outer surface of the V_2_CT*_x_* was transformed into amorphous VO*_x_* (a‐VO*_x_*) layers that conformally coated the parent V_2_C MXene. The remarkable synergistic properties arising from the combination of crystalline V_2_C and a‐VO*_x_* resulted in highly efficient Na^+^ storage performance. Specifically, the a‐VO*_x_* layer facilitated rapid and reversible Na^+^ insertion/extraction by offering abundant vacancies and opened pathways within its amorphous structure. Also, the V_2_C functioned as a conductive and robust framework, efficiently facilitating ion/electron transfer while preserving structural integrity. As a result, when used as a cathode for SIBs, the a‐VO*_x_*/V_2_C exhibited a substantial capacity of 307 mAh g^−1^ at 0.05 A g^−1^ and exceptional rate performance with capacity up to 96 mAh g^−1^ at 2 A g^−1^, outperforming its crystalline counterpart by a significant margin (Figure 9B). Recently, Yu et al. reported that crystalline WSe_2_ nanosheets grew in situ within atomically amorphous W–P clusters through controlled phosphorylation and selenization, resulting in an amorphous/crystalline heterostructure encapsulated in carbon nanosheets, which were then used as the anode in SIBs.^[^ ^127^ ^]^ As illustrated in Figure 9C, compared to amorphous W–P clusters and crystalline WSe_2_, the amorphous/crystalline W–P/WSe_2_ structure significantly enhanced interfacial charge redistribution and sodium interactions. Through P─Se bond coupling, the amorphous W–P clusters continued to uphold a stable W–P/WSe_2_ amorphous/crystalline heterostructure with the WSe_2_ nanosheet‐reconfigured quantum dots during prolonged cycling, which further enriched their interfaces, resulting in rapid and stable sodium‐ion storage.

In addition, Mukhopadhyay and team engineered Si nanowires with a crystalline core and an amorphous shell, demonstrating precise control over the thickness of the amorphous Si (a‐Si) shell surrounding the crystalline Si (c‐Si) core through careful manipulation of the deposition process.^[^ ^47^ ^]^ When Si nanowires with amorphous/crystalline heterostructure were used as anodes for SIBs, the optimized electrode achieved an impressive reversible Na^⁺^ capacity of 390 mAh g^−1^. Notably, both computational analyses and experimental evidence indicated that the primary contribution to the Na^⁺^ capacity originated from the bulk of the a‐Si shell, while the more conductive c‐Si core acts as a secondary “current collector” for the a‐Si shell. Moreover, Huu et al. synthesized orthorhombic sodium superionic conductor Fe_2_(MoO_4_)_3_ nanosheets featuring an amorphous–crystalline core–shell structure using a simple low‐temperature water‐vapor assisted solid‐phase reaction.^[^ ^128^ ^]^ When these nanosheets were used as the cathode in SIBs, they achieved a specific capacity of 90.2 mAh g^−1^ after 100 cycles and 87.8 mAh g^−1^ after 1000 cycles. The improved cycling performance could be attributed to the amorphous layer of the Fe_2_(MoO_4_)_3_ nanosheet, which featured a well‐organized 3D stacking structure and isotropy, allowing it to accommodate significant volume changes during the sodium ion insertion and extraction processes. In another study, Zhao and co‐workers prepared amorphous/crystalline MoO_2_ (a/c‐MoO_2_) by adjusting the volume ratio of the reaction medium in a one‐pot solvothermal reaction.^[^ ^55^ ^]^ Theoretical simulations indicated that electrons redistribute at the heterointerfaces of a/c‐MoO_2_, creating an inherent driving force that facilitates the adsorption of charge carriers and enhances electron/ion transfer efficiency. As shown in Figure 9D, the a/c‐MoO_2_ heterostructure exhibited better electrochemical activity and higher Na^+^ storage capacity than either a‐MoO_2_ or c‐MoO_2_, which is further verified in Figure 9E,F. The reversible discharge profiles at 0.25 A g^−^¹ (Figure 9E) intuitively proved that a/c‐MoO_2_ exhibited higher Na^+^ storage capacity (252.9 mAh g^−^¹) than a‐MoO_2_ (191.2 mAh g^−^¹) and c‐MoO_2_ (156.9 mAh g^−^¹). Additionally, the specific capacity, rate capability, and cycle performance of a/c‐MoO_2_ were notably superior to those of both a‐MoO_2_ and c‐MoO_2_ (Figure 9F).

In addition, Chen et al. utilized cellulose paper (CP) as the sole carbon source and K_2_FeO_4_ as the activator to synthesize porous graphitic/amorphous heterophase carbonized cellulose paper (PG‐CCP) featuring a lotus‐leaf‐like surface microstructure.^[^ ^129^ ^]^ Benefiting from the synergistic properties of graphitic and amorphous carbon, including a remarkable electrical conductivity of 14.3 S cm^−1^ and a large surface area of 372 m^2^ g^−1^. The PG‐CCP was directly employed as a freestanding, binder‐free anode, demonstrating excellent performance in SIBs with high specific capacities of 193 mAh g^−1^ at 100 mA g^−1^ and exceptional long‐term cycling stability over 1000 cycles.

#### 3.1.3 Potassium‐Ion Batteries (PIBs)

PIBs are being explored as another alternative to LIBs because of the abundant availability of K resources.^[^ ^130^ ^]^ However, K^+^ has a larger ionic radii, measuring 0.138 nm, than both Li^+^ (0.076 nm) and Na^+^ (0.106 nm), which presents a considerable challenge in finding suitable K‐ion host materials for PIBs.^[^ ^131^ ^]^ Introducing AC‐HNMs as an electrode is a good option to overcome the above challenges. The amorphous/crystalline heterostructure offers improved charge transport and enhanced intrinsic electronic conductivity by creating a built‐in electric field at the heterointerface.^[^ ^132^, ^133^ ^]^ Furthermore, the heterostructures provide a larger electrode–electrolyte contact area, which facilitates K^+^ diffusion at the interface and, in turn, enhances electrode reaction kinetics.^[^ ^42^ ^]^

**Figure 10:**
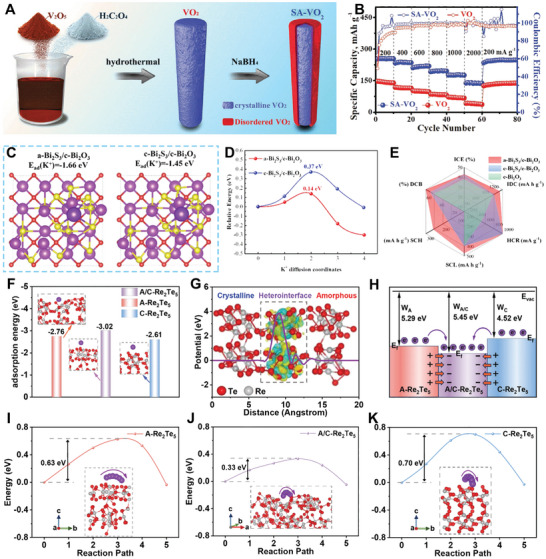
A) Schematic of the fabrication of SA‐VO_2_. B) Rate performance of SA‐VO_2_ and VO_2_ at different current densities. Reproduced with permission.^[^ ^134^ ^]^ Copyright 2020, Wiley‐VCH. C) A structural model of possible adsorption sites with K^+^ adsorption energy on a‐Bi_2_S_3_/c‐Bi_2_O_3_ and c‐Bi_2_S_3_/c‐Bi_2_O_3_. G) Comparison of the energy barrier of K^+^ diffusion in a‐Bi_2_S_3_/c‐Bi_2_O_3_ and c‐Bi_2_S_3_/c‐Bi_2_O_3_. E) Comparison results of initial coulombic efficiency (ICE), initial discharged capacity (IDC), highest current rate (HCR), low‐rate specific capacity (SCL), high‐rate specific capacity (SCH), and the proportion of capacitance contribution (PCC) with a‐Bi_2_S_3_/c‐Bi_2_O_3_, c‐Bi_2_S_3_/c‐Bi_2_O_3_, and c‐Bi_2_O_3_. Reproduced with permission.^[^ ^132^ ^]^ Copyright 2024, The Royal Society of Chemistry. F) K adsorption energy of A‐Re_2_Te_5_, A/C‐Re_2_Te_5_, and C‐Re_2_Te_5_. G) Charge density difference of A/C‐Re_2_Te_5_. H) Work function and formation process of the built‐in electric field of A‐Re_2_Te_5_, A/C‐Re_2_Te_5_, and C‐Re_2_Te_5_. Energy barriers for K diffusion in I) A‐Re_2_Te_5_, J) A/C‐Re_2_Te_5_, and K) C‐Re_2_Te_5_. Reproduced with permission.^[^ ^133^ ^]^ Copyright 2024, Wiley‐VCH.

In 2020, Lu's team introduced an interfacial engineering approach by inducing surface amorphization in VO_2_ nanorods, resulting in the formation of crystalline core/amorphous shell heterostructures (SA‐VO_2_, **Figure** **10**A).^[^ ^134^ ^]^ These structures showed great potential as anodes for PIBs, offering excellent K^+^ storage performances. Specifically, the crystalline core of VO_2_ interacted with its oxygen‐rich, defective amorphous shell to form a robust heterostructure interface that created a built‐in electric field, enabling fast interfacial charge transfer. Density functional theory (DFT) calculations revealed the presence of additional active sites near the heterointerfaces that exhibited a strong attraction to charge storage and possessed a low K^+^ diffusion barrier. As a result, the SA‐VO_2_ electrode demonstrated impressive reversible specific capacities of 253.1, 233.1, 211.3, 192.3, 176.3, and 141.4 mAh g^−1^ at current densities of 200, 400, 600, 800, 1000, and 2000 mA g^−1^, significantly surpassing the specific capacity of crystalline VO_2_ at the same current densities (Figure 10B).

In a separate study, Yang et al. developed a novel bismuth‐based anode material with an amorphous/crystalline heterostructure for PIBs by in situ growing amorphous Bi_2_S_3_ nanoparticles on 2D Bi_2_O_3_ nanosheets (a‐Bi_2_S_3_/c‐Bi_2_O_3_) using a precipitation method.^[^ ^132^ ^]^ Theoretical calculations revealed that the amorphous/crystalline heterostructure could enhance the adsorption energy and reduce the diffusion barrier of K^+^ (Figure 10C,D). Notably, the best K^+^ storage performances were achieved when the a‐Bi_2_S_3_/c‐Bi_2_O_3_ was used as the anode for PIBs compared to c‐Bi_2_S_3_/c‐Bi_2_O_3_, and c‐Bi_2_O_3_ (Figure 10E).

Very recently, Wu et al. prepared a novel superstructure consisting of amorphous/crystalline Re_2_Te_5_ anchored on an MXene substrate (A/C‐Re_2_Te_5_/MXene), which was used as an anode for PIBs.^[^ ^133^ ^]^ As shown in Figure 10F, the construction of amorphous/crystalline heterointerfaces enhanced the adsorption energy for K^+^ ions, which facilitated the ion supply for the intercalation reaction. Additionally, there was substantial charge accumulation and depletion at the heterointerfaces (Figure 10G), which promotes the generation of a built‐in electric field, thereby enhancing K^+^ adsorption/diffusion at the heterointerfaces. The work function analysis exhibited excellent electron trapping ability at the heterointerfaces of A/C‐Re_2_Te_5_ (Figure 10H), further confirming the generation of a built‐in electric field at the amorphous/crystalline heterointerfaces of A/C‐Re_2_Te_5_. Figure 10I–K further demonstrates the enhanced K^+^ diffusion kinetics in A/C‐Re_2_Te_5_.

In another study, Li et al. introduced a study on the utilization of layered potassium vanadium oxides, consisting of dual phases of amorphous K_0.25_V_2_O_5_·*n*H_2_O and crystalline V_2_O_5_ (referred to as ac‐K*_x_* V_2_O_5_), as an anode for aqueous potassium ion microbatteries.^[^ ^48^ ^]^ ac‐K*_x_* V_2_O_5_ with an amorphous/crystalline heterostructure featured amorphous K_0.25_V_2_O_5_·*n*H_2_O, which formed a molecular column, creating novel ion diffusion pathways and accommodating sites while preserving a large interfacial distance in the crystalline V_2_O_5_ bilayer (10.8 Å). This unique amorphous/crystalline heterostructure in ac‐K*_x_* V_2_O_5_ significantly enhanced the intercalation of hydrated K^+^. As a result, the resulting aqueous potassium‐ion microbatteries, featuring an ac‐K*_x_* V_2_O_5_ anode and crystalline K*_x_* MnO_2_·*n*H_2_O cathode, assembled on interdigital‐patterned nonporous metal current microcollectors, demonstrated an extraordinary energy density of 103 mWh cm^−3^.

#### 3.1.4 Zinc‐Ion Batteries (ZIBs)

**Figure 11:**
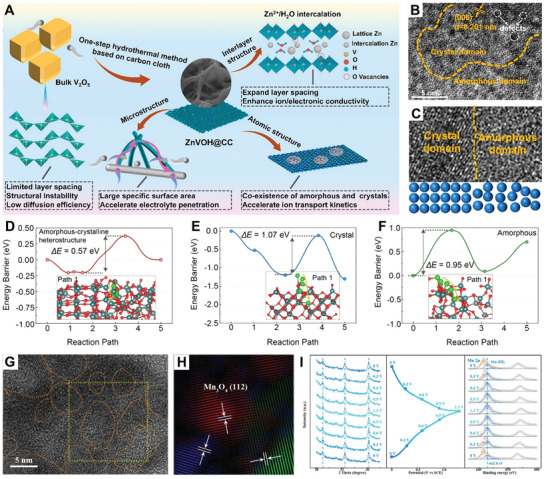
A) Schematic of the fabrication of ZnVOH@CC. B) TEM images of ZnVOH@CC. Reproduced with permission. C) Amorphous and crystalline domains heterostructure in ZnVOH@CC. Reproduced with permission.^[^ ^141^ ^]^ Copyright 2024, Elsevier. The energy barriers to Zn^2+^ diffusion along different pathways. D) Amorphous–crystalline structure, E) crystalline structure, and F) amorphous structure. Insets show the diffusion path. Reproduced with permission.^[^ ^54^ ^]^ Copyright 2023, Wiley‐VCH. G) HRTEM image and H) IFFT of Mn_3_O_4_‐A. I) Ex situ XRD, the ex situ XPS during the (dis)charging of Mn_3_O_4_‐A cathode at a current density of 0.5 A g^−1^. Reproduced with permission.^[^ ^53^ ^]^ Copyright 2022, Elsevier.

Zinc‐ion batteries (ZIBs) are considered superior to monovalent metal‐ion batteries, including LIBs, SIBs, and PIBs, in various respects including cost, safety, and reliability.^[^ ^135^, ^136^, ^137^, ^138^, ^139^ ^]^ AC‐HNMs have been applied in aqueous ZIBs to enhance their electrochemical performances.^[^ ^54^, ^140^, ^141^, ^142^, ^143^, ^144^ ^]^ Very recently, Sun et al. synthesized Zn*_x_* V_2_O_5_ *n*H_2_O with oxygen‐rich vacancies in situ on carbon cloth (ZnVOH@CC) using a one‐step hydrothermal method (**Figure** **11**A).^[^ ^141^ ^]^ Interestingly, numerous amorphous regions were visible alongside the distinct lattice striations of ZnVOH (Figure 11B), and the amorphous/crystalline heterointerface was also clearly observed (Figure 11C). These features provided more sites and diffusion pathways for Zn^2+^ storage. The ZnVOH@CC was employed as the cathode for AZIBs, which showed a high capacity of 464.9 mAh g^−1^ with a capacity retention rate of 98%. Similarly, Zhao et al. synthesized V_2_O_5_·1.6H_2_O (VOH) with an amorphous/crystalline heterostructure using a straightforward oxidation of the precursor VS_2_.^[^ ^145^ ^]^ The VOH cathode, with its triune crystal water–amorphous–crystalline hybrid structure, effectively accommodated stress and volume changes, reduced the strong electrostatic interaction between Zn^2+^ and the electrode material, expanded the Zn^2+^ transport pathway, and facilitated the rapid transmission of Zn^2+^, thus optimizing structural integrity and electrochemical performance. In another work, Wang and co‐workers synthesized a low crystalline vanadium oxide (VO‐E) with abundant amorphous/crystalline heterointerfaces using the electrochemical method, which then served as the cathode for AZIBs.^[^ ^54^ ^]^ As illustrated in Figure 11D–F, the amorphous/crystalline heterostructures displayed advantageous cation adsorption properties and lower energy barriers for ion diffusion when compared to both amorphous and crystalline counterparts. The VO‐E electrode demonstrated a remarkable discharge capacity of 516 mAh g^−1^ at 0.5 A g^−1^ (with a mass loading of 1.6 mg cm^−2^), surpassing the performance of its crystalline counterpart and many other reported V‐based cathode materials for AZIBs.

At the same time, Chen et al. synthesized MnCO_3_@amorphous MnO*_x_* (MnCO_3_@A‐MnO*_x_*) using a simple metathesis reaction and subsequent redox reaction, and utilized it as the cathode for AZIBs.^[^ ^142^ ^]^ The crystalline structure facilitated charge transfer and ion diffusion within the ordered lattice, offering high electronic conductivity and thermodynamic structural stability. The amorphous structure, with its short‐range atomic order and long‐range structural disorder, allowed for minimal volume expansion, making it well‐suited for long‐term stable cycling. As a result, the MnCO_3_@A‐MnO*_x_* electrode demonstrated a high discharge capacity of 316.9 mAh g^−^¹ at 0.1 A g^−^¹ and retained 71.6% of its capacity after 2000 cycles at 5.0 A g^−^¹.

#### 3.1.5 Magnesium‐Ion Batteries (MIBs)

MIBs hold significant promise as alternatives to LIBs, primarily due to their notable safety features, the ample availability of magnesium reserves, and high theoretical specific capacity.^[^ ^146^, ^147^, ^148^ ^]^ AC‐HNMs have also been used as electrode materials for MIBs to improve their electrochemical properties.^[^ ^53^, ^149^ ^]^ In 2022, Pan and collaborators utilized pretreated spinel Mn_3_O_4_ (Mn_3_O_4_‐A) as the cathode for aqueous MIBs.^[^ ^53^ ^]^ The amorphous ion channels and refined grains of spinel phase Mn_3_O_4_ were induced by pretreatment with Na_2_SO_4_ solution in a three‐electrode system. As shown in Figure 11G,H, the amorphous structure and the tiny grains are embedded in the amorphous structure in Mn_3_O_4_‐A. Based on the electrochemical kinetics and the ex‐situ XRD and XPS analyses (Figure 11I), the presence of amorphous ion channels reduced the interaction between Mg^2+^ ions and Mn_3_O_4_‐A, resulting in faster Mg^2+^ ion diffusion kinetics. Meanwhile, the refined grains exposed more active sites, promoting pseudocapacitive reactions between Mn(II) and Mn(III). As a result, the Mn_3_O_4_‐A cathode demonstrated a high specific discharge capacity of 98.9 mAh g^−1^ and an outstanding capacity retention rate of 99.4% after 2000 cycles at 0.2 A g^−1^.

In 2024, a WO_3_@WO_3−_ *_x_* S*_x_* crystalline@amorphous core–shell structure with both active core and shell was successfully created by introducing sulfur into metastable WO_3_ under controlled temperature conditions by Ding and co‐workers, and was utilized as the cathode for MIBs.^[^ ^150^ ^]^ In this design, the amorphous WO_3−_ *_x_* S*_x_* shell provided isotropic diffusion channels for Mg^2^⁺, allowing rapid ion migration to the interface where built‐in electric fields enhanced ion adsorption, thereby boosting specific capacity. Additionally, the amorphous shell relieved internal stress caused by repeated Mg^2+^ insertion/extraction in the crystalline WO_3_ core, extending cycle life. The incorporation of softer ions S^2−^ further reduced the strong interaction between the ionic lattice and Mg^2+^, thus improving Mg^2+^ storage kinetics. As a result, the WO_3_@WO_3−_ *_x_* S*_x_* cathode demonstrated outstanding cycling stability (83.3% capacity retention over 1000 cycles), impressive rate capability (88.5 mAh g^−1^ at 1000 mA g^−1^), and a high specific capacity (∼150 mAh g^−1^ at 50 mA g^−1^), nearly twice that of crystalline WO_3_ alone.

In addition, Zhang et al. designed a novel dual‐phase anode, consisting of amorphous Ge and crystalline Bi, for MIBs using a straightforward magnetron sputtering method.^[^ ^149^ ^]^ By incorporating the crystalline Bi phase into the amorphous Ge matrix, the defect formation energy for Mg^2+^ ion insertion was reduced, greatly improving the electrochemical reactivity of the amorphous Ge phase. The optimized amorphous Ge–crystalline Bi dual‐phase electrode achieved a high specific capacity of 847.5 mAh g^−^¹, significantly surpassing the specific capacity of crystalline Bi (~385 mAh g^−^¹).

### 3.2 Metal–Air Batteries

#### 3.2.1 Li–O_2_ Batteries (LOBs)

Among the advanced alternatives to LIBs, LOBs have garnered significant attention due to their impressive theoretical energy density (∼3500 Wh kg^−1^) that originates from the reversible reaction of lithium peroxide (O_2_ + 2Li^+^ + 2e^−^ ⇌ Li_2_O_2_).^[^ ^151^, ^152^, ^153^ ^]^ However, the sluggish kinetics of the reversible gas–solid reaction between O_2_ and Li_2_O_2_, along with the excessive accumulation of insulating discharge product Li_2_O_2_ on the cathode, resulted in low practical energy density, significant voltage hysteresis, undesirable polarization potential, and rapid capacity degradation in LOBs.^[^ ^154^, ^155^ ^]^ To achieve high‐performance LOBs, it is crucial to develop cathode catalysts that enhance oxygen electrochemical catalysis and efficiently regulate the formation and decomposition of Li_2_O_2_.^[^ ^156^ ^]^

AC‐HNMs have been reported as cathodes for LOBs and have shown excellent electrochemical performance. In 2021, Sun et al. used CoFeCe oxide with an amorphous/crystalline heterostructure as an effective electrocatalyst for the oxygen cathode in LOBs.^[^ ^52^ ^]^ Based on experimental findings and DFT analysis, the electrocatalytic activity of the amorphous/crystalline CoFeCe oxide in promoting the formation of decomposable Li_2_O_2_ was significantly enhanced due to synergistic interactions between oxide components, amorphous/crystalline heterostructure with minimized lattice mismatch, and improved the adsorption of the key intermediate LiO_2_. As a result, the assembled LOBs delivered a low overpotential of 0.95 V, a high initial discharge capacity of 12 340 mAh g^−1^, and significantly enhanced cycle stability (over 2900 h).

**Figure 12:**
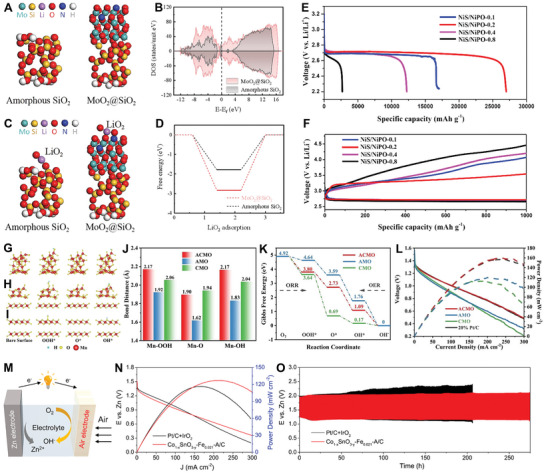
A) Optimized structures of amorphous SiO_2_ and MoO_2_@SiO_2_. B) Electronic density of states (DOS) for amorphous SiO_2_ and MoO_2_@SiO_2_. C) Optimized structures and D) adsorption energy diagrams for LiO_2_ adsorbed on amorphous SiO_2_ and MoO_2_@SiO_2_. Reproduced with permission.^[^ ^157^ ^]^ Copyright 2023, Wiley‐VCH. E) Discharge curves of LOBs with NiS/NiPO cathodes at 200 mA g^−1^. F) Discharge/charge profiles of LOBs with NiS/NiPO cathodes at 200 mA g^−1^ with a cut‐off capacity of 1000 mAh g^−1^. Reproduced with permission.^[^ ^158^ ^]^ Copyright 2023, Wiley‐VCH. Surface model with intermediates: G) AMO, H) ACMO, and I) CMO. J) Bond distance of three types of Mn─O bonds. L) Gibbs free energy diagram at 0 V. Reproduced with permission.^[^ ^165^ ^]^ Copyright 2022, Wiley‐VCH. M) Schematic of ZABs configuration. O) Polarization and power density curves of Co_1−_ *_x_* SnO_3−_ *_y_*‐Fe_0.021_‐A/C compared with Pt/C + IrO_2_ catalyst. P) Cyclic stability of ZABs at 5 mA cm^−2^. Reproduced with permission.^[^ ^106^ ^]^ Copyright 2023, American Chemical Society.

Recently, Sun et al. also reported a MoO_2_@coal gangue with amorphous/crystalline heterostructure extracted from mine solid waste as an electrocatalyst for the LOBs cathode.^[^ ^157^ ^]^ The amorphous SiO_2_ present on the surface of the coal gangue served as the substrate material, enhancing the structural stability of the MoO_2_@coal gangue. The results of the DFT calculation showed that N‐doped MoO_2_ nanoparticles have high electron conductivity and strong LiO_2_ absorption (**Figure** **12**A–D). Therefore, LOBs assembled with the MoO_2_@coal gangue cathode possessed excellent rate capacity (9748 mAh g^−1^) and durable cycle stability (∼2200 h).

In another study, an amorphous/crystalline heterostructure composed of amorphous nickel phosphate and crystalline nickel sulfide (NiS/NiPO) through the sulfurization of a hydrothermally generated precursor was constructed by Li and co‐workers.^[^ ^158^ ^]^ By carefully regulating the composition of the heterostructure, the electronic structure of the NiS/NiPO was optimized, enabling precise control over the growth path and morphology of the discharge products in LOBs. Specifically, NiS/NiPO with an optimized electronic structure was capable of promoting the deposition of a highly porous and interconnected Li_2_O_2_ structure, enriched with Li_2_O_2_–electrolyte interfaces. Moreover, abundant active sites were created on the NiS/NiPO through charge redistribution, facilitating uniform nucleation and the growth of Li_2_O_2_. As a result, the LOBs demonstrated an impressive high discharge capacity of 27003.6 mAh g^−1^ at 200 mA g^−1^ (Figure 12E) and a low charge overpotential of 0.58 V at 1000 mAh g^−1^ (Figure 12F).

#### 3.2.2 Zn–Air Batteries (ZABs)

Rechargeable ZABs are considered to be promising EES devices because of their notable advantages, including high theoretical energy density (~1370 Wh kg^−1^, excluding oxygen), high safety, and the low cost of abundant zinc ore (20 times less expensive than lithium), and the ease of zinc's preparation and storage.^[^ ^13^, ^159^, ^160^ ^]^ ZABs involve two important reactions at their air electrodes: oxygen evolution (OER) during charging and oxygen reduction (ORR) during discharge.^[^ ^161^, ^162^, ^163^ ^]^ However, the sluggish kinetics of the ORR and OER on air cathodes cause unsatisfactory battery output performances and charging characteristics, posing a significant barrier to the development of ZABs.^[^ ^10^, ^164^ ^]^ Therefore, the demand for efficient electrocatalysts capable of accelerating the ORR/OER kinetics remains critical to improving the activity and stability of ZABs.

Constructing amorphous/crystalline heterostructures in electrocatalysts can improve their catalytic reaction kinetics. For example, Zhou and co‐workers designed a layered MnO_2_ with amorphous/crystalline heterostructure (ACMO) as an effective electrocatalyst for the oxygen cathode of ZABs.^[^ ^165^ ^]^ As shown in Figure 12G–K, the DFT calculation results indicated the amorphous/crystalline heterostructure could optimize the adsorption of reactants and intermediates on the ACMO surface, accelerating the ORR/OER reaction kinetics. When the ACMO was assembled into ZABs, the devices achieved a high power density of 159.7 mW cm^−2^ at a current density of 239.1 mA cm^−2^ (Figure 12L), which was far superior to amorphous layered MnO_2_ (AMO) and well‐crystallized layered MnO_2_ (CMO) and comparable to commercial 20% Pt/C (159.2 mW cm^−2^).

In another study, Wang et al. reported an amorphous/crystalline ternary metal (oxy)hydroxide reconstructed by amorphous multi‐metallic sulfide (FeCoNiS*_x_*).^[^ ^166^ ^]^ Theoretical calculations indicated that the synergistic effect and electronic coupling of multimetallic active sites in the unique amorphous/crystalline heterointerfaces formed by self‐reconstruction promoted the OER kinetics. In addition, the migration of intermediate O* from Ni sites to Fe sites helped to break the limitation of the scaling relation, which further enhanced the OER activity. Therefore, the assembled ZABs achieved long‐life, and fast charging at high current density. Recently, Ye et al. carried out the tailoring of metal–oxygen bonds by designing amorphous/crystalline heterostructures, thereby significantly promoting the catalytic activity for OER/ORR.^[^ ^106^ ^]^ The introduction of crystal structures in amorphous CoSnO_3_ dominated the tailoring of metal–oxygen bond structures, which was further facilitated by the introduction of Fe─O bonds and abundant cobalt defects. As a result, the ZABs based on Co_1−_ *_x_* SnO_3−_ *_y_*‐Fe_0.021_‐A/C exhibited outstanding output power density and cycling performance (Figure 12M–O).

### 3.3 Lithium–Sulfur Batteries

Lithium–sulfur batteries (LSBs) are an attractive option for next‐generation battery systems because of the natural abundance of sulfur and a high theoretical specific energy of 2600 Wh kg^−1^, which is about five times higher than those of current LIBs.^[^ ^167^, ^168^, ^169^, ^170^ ^]^ Nevertheless, the practical application of LSBs has been impeded by the insulation of sulfur, high reaction energy barrier, slow redox reaction kinetics, and the detrimental shuttling effect of lithium polysulfides (LiPSs).^[^ ^171^, ^172^, ^173^ ^]^ It was found that the construction of amorphous/crystalline heterostructures can improve electronic and ionic conductivity and enhance the capture and catalytic conversion of lithium polysulfide in LSBs, which can significantly improve the performance of LSBs.^[^ ^59^, ^174^ ^]^

**Figure 13:**
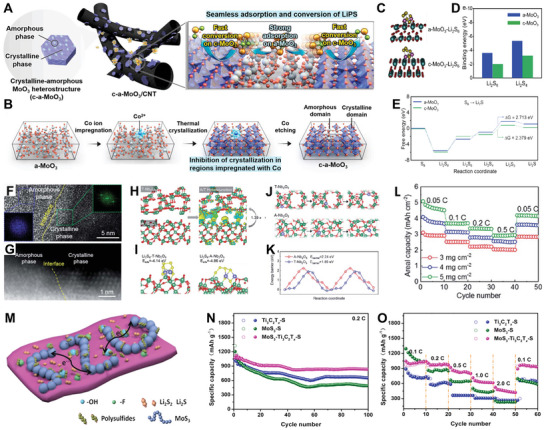
A) Atomic scale crystalline–amorphous MoO_3_ heterostructure. B) Preparation process of c–a‐MoO_3_. C) Optimized atomic configuration images for Li_2_S_6_ adsorption on a‐MoO_3_ and c‐MoO_3_. D) Calculated binding energies for various lithium polysulfide molecules on a‐MoO_3_ and c‐MoO_3_. E) Free energy evolution for conversion from S_8_ to Li_2_S on a‐MoO_3_ and c‐MoO_3_, respectively. Reproduced with permission.^[^ ^175^ ^]^ Copyright 2024, Elsevier. F) HRTEM image with insets showing the FFT patterns of the selected areas. G) AC‐HAADF‐STEM image of A/T‐Nb_2_O_5_. H) Calculated geometries of A‐Nb_2_O_5_ and T‐Nb_2_O_5_, as well as A/T‐Nb_2_O_5_ heterostructure, with differences in electron density. I) Optimized configuration of the Li_2_S_6_ adsorption on the surface of A‐Nb_2_O_5_ and T‐Nb_2_O_5_. J) Geometrical configurations and K) energy profiles of Li^+^ diffusion on A‐Nb_2_O_5_ and T‐Nb_2_O_5_ surfaces. L) Rate performances of the S‐A/T‐Nb_2_O_5_//Li‐A/T‐Nb_2_O_5_ full cell. Reproduced with permission.^[^ ^51^ ^]^ Copyright 2021, The Royal Society of Chemistry. M) Schematic illustration of the LiPS trapping and conversion process on the MoS_3_–Ti_3_C_2_T*_x_* heterostructures. N) Cycling performance and O) rate performances of MoS_3_–S, Ti_3_C_2_T*_x_*–S, and MoS_3_–Ti_3_C_2_T*_x_*–S cathodes. Reproduced with permission.^[^ ^177^ ^]^ Copyright 2021, Elsevier.

Very recently, Lee et al. developed a crystalline–amorphous MoO_3_ heterostructure (c‐a‐MoO_3_) as a cathode host for LSBs by partially inhibiting the phase transition of a‐MoO_3_ through the addition of metal ions (**Figure** **13**A,B).^[^ ^175^ ^]^ The design of the heterostructure enhanced overall sulfur conversion efficiency by combining the high adsorption capacity of amorphous MoO_3_ with the rapid conversion kinetics of crystalline MoO_3_. As shown in Figure 13C,D, a‐MoO_3_ exhibited higher adsorption energy than c‐MoO_3_ for Li_2_S_4_ and Li_2_S_6_. Furthermore, c‐MoO_3_ exhibited a lower energy barrier for Li^+^ diffusion and Li_2_S_2_ formation compared to a‐MoO_3_, which indicated that c‐MoO_3_ possessed superior transformation kinetics (Figure 13E). Accordingly, the LSBs based on c‐a‐MoO_3_ showed superior performance, achieving a discharge capacity of up to 1520 mAh g^−1^ at 0.05 C, while maintaining a high capacity of 1048 mAh g^−1^ even at 4 C, and demonstrating stable cycling performance.

In another study, Wang and co‐workers designed a niobium oxide matrix with amorphous/crystalline heterointerfaces (A/T‐Nb_2_O_5_) as a host material for both sulfur and lithium electrodes in LSBs by simply regulating the calcination temperature.^[^ ^51^ ^]^ As shown in Figure 13F, a distinct boundary separates the amorphous phase (left) from the crystalline phase (right), identifiable by disordered patterns on the left and clear lattice stripes on the right, which was further confirmed by FFT patterns in selected regions. More importantly, aberration‐corrected high‐angle annular dark‐field scanning transmission electron microscopy (AC‐HAADF‐STEM) images (Figure 13G) also revealed a distinct interface between the amorphous and crystalline Nb_2_O_5_, characterized by the disordered atomic arrangement in the amorphous region and ordered atomic arrangement in the crystalline region. The DFT calculation results showed that the combination of crystalline Nb_2_O_5_ (T‐Nb_2_O_5_) and amorphous Nb_2_O_5_ (A‐Nb_2_O_5_) could be highly promising, to simultaneously realize shuttle inhibition and improved kinetics (Figure 13H–K). On the cathode side, the A/T‐Nb_2_O_5_ enhanced its intrinsic conductivity by generating a significant built‐in electric field from the heterointerfaces. The A/T‐Nb_2_O_5_ combined the high lithium polysulfide adsorption capacity of A‐Nb_2_O_5_ with the low lithium‐ion mobility barrier of T‐Nb_2_O_5_, effectively suppressing the shuttle effect and enhancing sulfur reaction kinetics. On the anode side, the A/T‐Nb_2_O_5_ inhibits the growth of lithium dendrites by reducing the generation of local currents due to its unique lithiophilicity and porous structure. As a result, the fabricated S‐A/T‐Nb_2_O_5_//Li‐A/T‐Nb_2_O_5_ battery demonstrated reversible cycling at high current rates and sulfur loadings, achieving a high areal capacity of 2.92 mAh cm^−2^ at 0.5 C with a sulfur loading of 5 mg cm^−2^ (Figure 13L).

In addition, a core–shell nanobox with a crystalline porous MnO core and amorphous TiO_2_ shell (MnO@TiO_2_) was synthesized via the wet‐chemical method by Yang and co‐workers.^[^ ^176^ ^]^ The MnO@TiO_2_ structure featured a core–shell design that reduced volume expansion during the charge/discharge process, with amorphous TiO_2_ shells providing strong chemical interactions that facilitated polysulfide dissolution in the electrolyte, and porous crystalline MnO cores offering robust sulfur anchoring and efficient polysulfide adsorption. By integrating MnO@TiO_2_ with RGO nanosheets, its electrical conductivity was enhanced, resulting in MnO@TiO_2_/RGO‐S nanocomposites that served as a promising high‐capacity cathode material for LSBs. In another study, Chen et al. prepared a multifunctional amorphous/crystalline heterostructure (MoS_3_–Ti_3_C_2_T*_x_*) consisting of functionalized MXene (Ti_3_C_2_T*_x_*) and amorphous MoS_3_ by a scalable electrostatic self‐assembly method.^[^ ^177^ ^]^ As shown in Figure 13M, the LiPSs demonstrated strong chemical interactions with amorphous MoS_3_, leading to their conversion into solid Li_2_S_2_/Li_2_S. Meanwhile, the crystalline Ti_3_C_2_T*_x_* offered an ample surface area which alleviated volume expansion and facilitated the effective conversion of LiPSs. Additionally, the functional groups on the Ti_3_C_2_T*_x_* surface served as adsorption centers for capturing LiPSs. The LSBs with the MoS_3_–Ti_3_C_2_T*_x_* heterostructure exhibited a higher specific capacity of 836 mAh g^−1^ after 100 cycles at 0.2 C and an outstanding rate capability of 463 mAh g^−1^ at 2 C (Figure 13N,O).

### 3.4 Supercapacitors

Supercapacitors (SCs) have attracted growing interest because of their distinct advantages over other energy storage devices, including high power density, superior cycling stability, and fast charge/discharge rate.^[^ ^18^, ^178^, ^179^, ^180^ ^]^ Nevertheless, the energy density of SCs remains lower than that of batteries, which restricts their widespread commercial adoption for EES applications.^[^ ^181^, ^182^, ^183^ ^]^ The development of high‐performance electrode materials has become the key goal for enhancing the energy density of SCs.^[^ ^184^, ^185^, ^186^, ^187^ ^]^ Among them, AC‐HNMs show great promise as advanced electrode materials, leveraging abundant active sites at phase boundaries, the synergistic effect between different phases, and the built‐in electric field generated at the amorphous/crystalline heterointerface, all contributing to improved electrochemical performances.^[^ ^41^, ^188^, ^189^ ^]^

**Figure 14:**
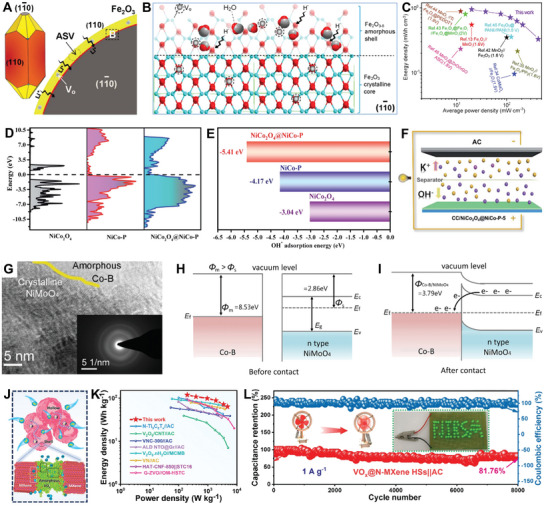
A) Illustration of a single ASV‐FO nanorod with exposed (110) surface plane and (1‐10) cross‐section plane. B) Atomic configurations of the selected area in the dashed box in (A). C) Ragone plots of the solid‐state ASV‐FO//V‐CO ASC. Reproduced with permission.^[^ ^190^ ^]^ Copyright 2018, Elsevier. D) The TDOS plots of NiCo_2_O_4_, NiCo‐P, and NiCo_2_O_4_@NiCo‐P. E) Adsorption energy of OH^−^ on NiCo_2_O_4_, NiCo‐P, and NiCo_2_O_4_@NiCo‐P. F) Schematic illustration of the HSC device. Reproduced with permission.^[^ ^91^ ^]^ Copyright 2022, The Royal Society of Chemistry. G) TEM images and SAED patterns of NiMoO_4_@Co–B. H,I) Energy band schematic of the Mott–Schottky heterojunction of the NiMoO_4_@Co–B before and after contacting. Ec: conduction band; Ev: valence band; Ef: Fermi level; Eg: bandgap; U: work function. Reproduced with permission.^[^ ^44^ ^]^ Copyright 2023, Elsevier. J) Charge/discharge schematic diagram of VO*_x_* @N‐MXene HSs. K) Ragone plots of sodium‐ion hybrid capacitor device and reported literature. L) Cycling stability of the device at 1 A g^−1^. Reproduced with permission.^[^ ^194^ ^]^ Copyright 2024, Wiley‐VCH.

In 2018, Sun et al. introduced a novel oxygen‐deficient Fe_2_O_3‐δ_ nanorod arrays with a distinctive crystalline core and amorphous shell heterostructure (ASV‐FO) via a simple NaBH_4_ treatment.^[^ ^190^ ^]^ This treatment could induce oxygen vacancies (V_o_) and form an amorphous surface layer on the surfaces of the Fe_2_O_3_ nanorods (**Figure** **14**A,B), with the concentration of NaBH_4_ controlling both the oxygen vacancy content and the thickness of the amorphous shell. The introduced V_o_ improved electronic conductivity, while the amorphous shell promoted rapid Li^+^ diffusion, and the crystalline/amorphous interfaces further enhanced charge storage capacity by providing additional storage sites. As a result, a flexible large‐scale asymmetric supercapacitor, comprised of oxygen‐deficient Co_3_O_4‐δ_ (V‐CO) nanosheet arrays as the cathode and ASV‐FO as the anode, exhibited a maximum energy density of 0.95 mWh cm^−3^ at 20 mW cm^−3^ (Figure 14C). In 2021, Ye et al. synthesized hybrid CoNiO_2_ nanowires with amorphous/crystalline heterostructures on nickel foam.^[^ ^191^ ^]^ The crystalline phase of the CoNiO_2_ contributed to low internal energy and remarkable chemical stability, while the amorphous phase facilitated the rapid diffusion of exposed ions and accelerated charge transfer. This combination of crystalline and amorphous properties enhanced the overall performance of the hybrid nanowires. Used as the cathode in zinc‐ion hybrid supercapacitors (ZHSCs), the amorphous/crystalline CoNiO_2_ nanowires demonstrated a remarkable specific capacity of 300.2 mA g^−1^ at a current density of 0.5 A g^−1^, achieving an impressive energy density of 499.2 Wh kg^−1^ in the hybrid CoNiO_2_//Zn ZHSC configuration. In 2024, Li et al. prepared an amorphous/crystalline nickel manganese phosphate octahydrate heterostructure supported on nickel foam through a simple hydrothermal method followed by thermal treatment under an argon atmosphere.^[^ ^192^ ^]^ In this structure, the amorphous portions promoted ion diffusion and reaction kinetics, while the crystalline components provided both active sites and mechanical stability. Subsequently, an aqueous asymmetric supercapacitor was constructed using nickel manganese phosphate octahydrate as the cathode and active carbon as the anode, achieving an impressive energy density of 66.22 Wh kg^− 1^ at a power density of 400 W kg^−1^.

In addition, Gao and co‐workers constructed amorphous/crystalline heterostructures by encapsulating crystalline NiCo_2_O_4_ nanowires, which were anchored on carbon cloth, with ultrathin nanosheets of amorphous NiCo‐(HPO_4_)_2_ H_2_O (NiCo‐P), resulting in the structure designated as CC/NiCo_2_O_4_@NiCo‐P.^[^ ^91^ ^]^ The DFT results confirmed that the CC/NiCo_2_O_4_@NiCo‐P effectively promoted electron transfer, increased the electrochemical reaction active sites, and enhanced the adsorption capacity for OH^−^ through synergistic interactions at the amorphous/crystalline heterointerfaces, facilitating rapid redox reaction kinetics (Figure 14D,E). When a hybrid supercapacitor (HSC) was assembled with CC/NiCo_2_O_4_@NiCo‐P as the anode and activated carbon as the cathode (Figure 14F), it demonstrated a high energy density of 54.83 Wh kg^−1^ at a high power density of 682.53 W kg^−1^. In a similar study, Mule et al. reported the deposition of amorphous ultrathin nickel–manganese hydroxide (NiMn) nanorods onto conducting crystalline hydrated nickel–molybdenum oxide (NiMo) nanorods (NRs) anchored on nickel foam, resulting in amorphous/crystalline heterostructures (NiMo NRs@NiMn NSs).^[^ ^193^ ^]^ The synergistic interaction between the conducting crystalline core and the amorphous shell in NiMo NRs@NiMn NSs significantly facilitated the charge dynamics, which compensated for the shortcomings of the crystalline hydrated nickel–molybdenum oxide alone. The assembled HSC device exhibited high energy and power densities, along with excellent cycle stability.

Recently, Hou et al. constructed amorphous/crystalline heterostructures by coating amorphous Co–B nanosheets on crystalline NiMoO_4_ nanorods (NiMoO_4_@Co–B) via a facile chemical reduction method.^[^ ^44^ ^]^ As shown in Figure 14G, the amorphous/crystalline heterointerface could be clearly observed. When the intermetallic compound Co–B came into direct contact with the n‐type semiconductor NiMoO_4_, the difference in Fermi energy levels caused electrons to transfer from the NiMoO_4_ to Co–B, resulting in the establishment of a built‐in electric field (Figure 14H,I). The built‐in electric field reduced the reaction energy barriers, provided additional active storage sites, and effectively regulated the charge transfer kinetics. The amorphous Co–B buffer layer alleviated structural stress during the discharge process, facilitating high‐rate charge storage. The optimized NiMoO_4_@Co–B electrode exhibited a reversible specific capacity of 236.2 mAh g^−1^ at 0.5 A g^−1^ and maintained a high specific capacity retention of 72.5% at 20 A g^−1^.

In another study, Li and co‐workers achieved a partial conversion of crystalline CoGa_2_O_4_ into an amorphous phase via a controlled sulfurization process, followed by anchoring crystalline NiCo‐LDH onto amorphous/crystalline CoGa_2_O_4_‐S to construct freestanding 3D CoGa_2_O_4_‐S@NiCo‐LDH core–shell heterostructures with distinctivecrystalline/amorphous/crystalline heterointerfaces on carbon cloth.^[^ ^56^ ^]^ Atomic‐level sulfur doping in CoGa_2_O_4_ altered the microstructure and fine‐tuned the electronic configuration, partially converting the crystalline phase to an amorphous state, which enhanced OH^−^ diffusion rates and increased electrochemical activity. Meanwhile, the crystalline NiCo‐LDH shell layer facilitated rapid electron transfer. The resulting sandwich‐like flexible CoGa_2_O_4_‐S@NiCo‐LDH//AC asymmetric supercapacitor achieved an energy density of 58.2 Wh kg^−1^ at a power density of 850 W kg^−1^.

Very recently, Wen's group designed a novel hollow sphere hybrid structure by encapsulating amorphous vanadium oxide into crystalline N‐doped MXene (VO*_x_* @N‐MXene HSs) through a facile self‐assembly process.^[^ ^194^ ^]^ Machine learning and molecular simulations were used to analyze the charge/discharge processes, structural evolutions, and reaction pathways, demonstrating the effectiveness of the hollow sphere hybrid structure for sodium storage. This sophisticated hollow hybrid material harnessed the exceptional structural advantages of amorphous, crystalline, and hollow structures, enabling rapid reaction kinetics, reversible and fast Na^+^ insertion/extraction (Figure 14J). Notably, the assembled sodium‐ion hybrid capacitors demonstrated an impressive energy density of 198.3 Wh kg^−1^ (Figure 14K) and notable capacity retention of 81.76% after 8000 cycles (Figure 14L).

## 4 Challenges and Perspectives

This review summarizes recent progress in the preparation of AC‐HNMs and their application in EES. Various preparation methods, including one‐step and stepwise approaches, have been developed to manufacture different kinds of AC‐HNMs. The AC‐HNMs, characterized by excellent physical and chemical properties such as high electronic conductivity and exceptional thermal stability, show great potential for EES applications. Their effectiveness is largely due to the synergistic interactions between the distinct phases, the numerous active sites at the amorphous/crystalline heterointerfaces, and the built‐in electric fields formed at the heterointerfaces. **Table** **1** showcases representative AC‐HNMs and their applications in metal‐ion batteries, metal–air batteries, lithium–sulfur batteries, and supercapacitors, affirming their unique structures and outstanding properties. While various types of AC‐HNMs have been reported, research remains in an early stage, and several challenges still need to be addressed, as follows.

1. The directional synthesis of AC‐HNMs is particularly challenging due to the absence of long‐range order in the amorphous phase. Exploring the general synthetic methods of AC‐HNMs and effectively regulating structure and composition represents a critical direction for the future development of AC‐HNMs. The rapid advancement of artificial intelligence, particularly in machine learning and deep learning algorithms, can facilitate high‐throughput searching, reliable modeling, and theoretical predictions, thereby enabling more directed and efficient synthesis of AC‐HNMs.
2. The large‐scale production of AC‐HNMs remains a hurdle, as existing synthesis methods are often time‐consuming, energy‐intensive, and labor‐intensive. Moreover, the yield, quality, and uniformity of AC‐HNMs are inadequate for commercial and industrial demands. As a result, optimizing existing synthesis techniques and developing new, versatile, eco‐friendly, and scalable methods is urgently needed to facilitate the high‐quality, cost‐effective mass production of AC‐HNMs.
3. The formation mechanisms involved in synthesizing AC‐HNMs are lacking and ambiguous, which limits the variety and yield of these materials. Traditional characterization techniques struggle to reveal atomic arrangements and chemical bonding at specific interfacial sites. However, utilizing cutting‐edge characterization technologies, such as AC‐HAADF‐STEM and high‐resolution XAS, could offer valuable insights into growth processes. Moreover, in situ/operando characterization tools, including in situ TEM and XRD, are expected to shed light on the dynamic reactions occurring during the growth of AC‐HNMs.
4. Investigating the relationship between phase‐dependent properties and applications is critical, yet synthesizing nanomaterials with identical or similar morphologies across crystalline, amorphous, and amorphous/crystalline phases presents a challenge. For example, even when efforts are made to minimize the influence of other structural features, producing crystalline nanomaterials, amorphous nanomaterials, and AC‐HNMs with the same or similar morphologies remains a complex task, hindering the study of their phase‐dependent properties and applications.
5. Understanding of the reaction mechanisms of AC‐HNMs in specific EES applications remains quite limited, as most current studies overlook the evolution of surface and interface structures during electrochemical reactions. Therefore, it is crucial to prioritize detailed investigations into changes in phase structure, chemical composition, valence states, and local structures through in‐situ or quasi‐in situ characterization techniques. Additionally, combining electrochemical testing with theoretical calculations is necessary to clarify electrode dynamics and interfacial charge transport, which can ultimately reveal the underlying reaction mechanisms.
6. Theoretical calculations play an important role in exploring the formation mechanism and reaction mechanism of AC‐HNMs. However, there is a lack of theoretical understanding of the formation and reaction mechanism of the amorphous phase. This challenge arises from the complex and disordered arrangement of atoms in the amorphous phase, which makes it difficult to construct convincing models and accurately simulate their behaviors. The precise construction of theoretical models that show good accordance with the realistic structures of AC‐HNMs is important to conducting further theoretical studies.

**Table 1:** Typical AC‐HNMs as electrode materials for EES applications. (a and c represent amorphous and crystalline phases, respectively).

Application	Material	Media	Performances	Refs.
LIBs	a/c‐MnO_2_	Nonaqueous	1210 mAh g^−1^ at 0.1 A g^−1^/only fading 1% after 2500 cycles at 1 A g^−1^	[50]
LIBs	c‐ZnCo_2_O_4_/a‐Co–B	Nonaqueous	843 mAh g^−1^ at 0.5 A g^−1^/766 mAh g^−1^ after 1000 cycles at 10 A g^−1^	[118]
LIBs	c‐Fe_2_O_3_@a‐NiMoO_4_‐S	Nonaqueous	1001 mAh g^−1^ after 100 cycles at 0.1 A g^−1^/766 mAh g^−1^ after 1000 cycles at 10 A g^−1^	[195]
LIBs	c‐Si/a‐SiO_2_	Nonaqueous	1200 mAh g^−1^ at 0.2 C/≈1130 mAh g^−1^ after 500 cycles at 0.2 C	[117]
LIBs	C/c‐Cu_9_S_5_–a‐MoS*_x_*	Nonaqueous	561 mAh g^−1^ at 2 A g^−1^/445 mAh g^−1^ at 6 A g^−1^/376 mAh g^−1^ after 3600 cycles at 2 A g^−1^	[113]
LIBs	a/c‐V_2_O_5_	Aqueous	262.71 mAh g^−1^ at 0.5 A g^−1^/123.76 mAh g^−1^ at 10 A g^−1^	[115]
SIBs	a/c‐MoO_2_	Nonaqueous	307 mAh g^−1^ at 0.1 A g^−1^/86% reversible capacity over 3000 cycles at 1 A g^−1^	[55]
SIBs	a/c‐Fe_2_(MoO_4_)_3_	Nonaqueous	87.8 mAh·g^−1^ after 1000 cycles at 1 C/64.8 mAh·g^−1^ at 10 C	[128]
SIBs	c‐Si@a‐Si	Nonaqueous	390 mAh g^−1^ at 0.1 C	[47]
SIBs	a/c‐V_2_O_3‐_ *_x_* @C‐HMCS	Nonaqueous	261 mAh g^−1^ at 1 A g^−1^ over 2000 cycles/192 mAh g^−1^ over 6000 cycles at 10 A g^−1^	[196]
SIBs	c‐Co_4_S_3_ *@*NSC/a‐MoS_3_	Nonaqueous	594 mAh g^−1^ at 100 cycles 0.5 A g^−1^/382 mAh g^−1^ at 10 A g^−1^	[197]
SIBs	a‐VO*_x_*/c‐V_2_C	Nonaqueous	307 mAh g^−1^ at 50 mA g^−1^/96 mAh g^−1^ at 2000 mA g^−1^/54 mAh g^−1^ after 1800 cycles at 2000 mA g^−1^	[126]
SIBs	a‐W‐P/c‐WSe_2_/C	Nonaqueous	460.8 mAh g^−1^ at 0.1 A g^−1^/217.4 mAh g^−1^ after 5000 cycles at 10 A g^−1^	[127]
PIBs	a/c‐VO_2_	Nonaqueous	288.3 mAh g^−1^ at 50 mA g^−1^/141.4 mAh g^−1^ at 2000 mA g^−1^/86% capacity retention over 500 cycles at 500 mA g^−1^	[134]
PIBs	a/c‐Re_2_Te_5_/MXene	Nonaqueous	350.4 mAh g^−1^ after 200 cycles at 0.2 A g^−1^/162.5 mAh g^−1^ at 20 A g^−1^/186.1 mAh g^−1^ over 5000 cycles at 5 A g^−1^	[133]
PIBs	a‐Bi_2_S_3_/c‐Bi_2_O_3_	Nonaqueous	289 mAh g^−1^ at 200 A g^−1^ after 60 cycles/226 mAh g^−1^ at 1000 mA g^−1^	[132]
PIBs	c‐TiO_2_@a‐MoS_3_@NC	Nonaqueous	345 mAh g^−1^ after 200 cycles at 0.5 A g^−1^/104 mAh g^−1^ at 5 A g^−1^/144 mAh g^−1^ at 2 A g^−1^ for 1000 cycles	[198]
PIBs	a‐K_0.25_V_2_O_5_ *n*H_2_O/c‐V_2_O_5_	Aqueous	715 mAh cm^−3^ at 5 mV s^−1^/153 mAh cm^−3^ at 1000 mV s^−1^	[48]
ZIBs	a/c‐MnO_2_	Aqueous	257 mAh g^−1^ at 0.1 A g^−1^/not degrade after 100 cycles at 0.5 A g^−1^	[199]
ZIBs	a/c‐V_2_O_5_	Aqueous	516 mAh g^−1^ at 0.1 A g^−1^/85.5% capacity retention after 5000 cycles	[54]
ZIBs	a/c‐Zn*_x_* V_2_O_5_⋅*n*H_2_O@CC	Aqueous	464.9 mAh g^−1^ at 0.1 A g^−1^/213 mAh g^−1^ at 10 A g^−1^/96.2% capacity retention after 2000 cycles at 5 A g^−1^	[141]
ZIBs	c‐MnCO_3_@a‐MnO*_x_*	Aqueous	316.9 mAh g^−1^ at 0.1 A g^−1^/71.6% capacity retention after 2000 cycles at 5 A g^−1^	[142]
ZIBs	CC/a‐V_2_O_5_@c‐Ti_3_C_2_T*_x_*	Aqueous	567 mAh g^−1^ at 0.1 A g^−1^/213 mAh g^−1^ at 10 A g^−1^/96.2% capacity retention after 1000 cycles at 5 A g^−1^	[144]
MIBs	a/c‐Mn_3_O_4_	Aqueous	98.9 mAh g^−1^ at 0.1 A g^−1^/99.4% capacity retention after 2000 cycles at 0.2 A g^−1^	[53]
MIBs	c‐Ge/a‐Bi	Nonaqueous	847.5 mAh g^−1^ at 50 mA g^−1^/435.5 mAh g^−1^ at 2000 mA g^−1^	[149]
MIBs	c‐WO_3_@a‐WO_3−_ *_x_* S*_x_*	Nonaqueous	150 mAh g^−1^ at 50 mA g^−1^/88.5 mAh g^−1^ at 1000 mA g^−1^/83.3% capacity retention after 1000 cycles at 1000 mA g^−1^	[150]
LOBs	a/c‐CoFeCe oxides	Aqueous	12 340 mAh g^−1^ at 100 mA g^−1^/cyclic stability over 2900 h	[52]
LOBs	c‐NiS/a‐NiPO@NF	Aqueous	27 003.6 mAh g^−1^ at 200 mA g^−1^/24 884.3 mAh g^−1^ at 400 mA g^−1^	[158]
LOBs	c‐MoO_2_@a‐coal gangue	Aqueous	9748 mAh g^−1^ at 100 mA g^−1^/endurable cycle stability (≈2200 h)	[157]
ZABs	a/c‐MnO_2_	Aqueous	733 mAh g^−1^ 20 mA g^−1^/cycled stably for 1000 cycles (≈17 days)	[200]
ZABs	a/c‐Pd_75.9_Mo_9_._4_Cr_8.9_W_5.8_/C	Aqueous	895 mAh g^−1^ at 5 mA g^−1^/ultralong durability of 329 h	[201]
ZABs	a/c‐NiFeMoO*_x_*	Aqueous	Stably cycled for up to 280 h	[58]
ZABs	a/c‐Co_1−_ *_x_* SnO_3−_ *_y_*‐Fe_0.021_	Aqueous	765 mAh g^−1^ at 5 mA g^−1^/long cycle stability for 275 h	[106]
ZABs	a/c‐FePSe_3_‐O	Aqueous	Stably cycled for up to 280 h	[99]
ZABs	c‐Co_2_P@a‐FePO_4_/C	Aqueous	852.36 mAh g^−1^ at 50 mA g^−1^	[49]
ZABs	c‐Co/a‐Co_3_O_4_/NC/RGO	Aqueous	198.7 mW cm^−2^ at 305.2 mA cm^−2^/increase from 0.78 V to 0.94 V for 58 h at 20 mA cm^−2^	[202]
LSBs	a/c‐Nb_2_O_5_	Nonaqueous	1291 mAh·g^−1^ at 0.1 C/600 mAh g^−1^ after 800 cycles at 0.5 C	[51]
LSBs	a/c‐MoO_3_	Nonaqueous	1520 mAh g^−1^ at 0.05 C/decay rate of 0.0713% over 500 cycles at 1 C	[175]
LSBs	c‐MnO@a‐TiO_2_/RGO	Nonaqueous	1451 mAh g^−1^ at 0.1 C/≈986 mAh g^−1^ at 0.2 C after 100 cycles	[176]
LSBs	a‐MoS_3_/c‐Ti_3_C_2_T*_x_*	Nonaqueous	1043 mAh g^−1^ at 200 mA g^−1^/568 mAh g^−1^ at 2 A g^−1^ after 1000 cycles	[177]
SCs	a/c‐Ni*_x_* Co*_y_* OOH	Aqueous	3720 F g^−1^ at 1 A g^−1^/2311 F g^−1^ at 20 A g^−1^	[67]
SCs	a/c‐CoNiO_2_	Aqueous	300.2 mAh g^−1^ at 0.5 A g^−1^/91.7% capacity retention after 5000 cycles at 30 mA cm^−2^	[191]
SCs	a/c‐Fe_2_O_3‐δ_	Aqueous	423 mF cm^−2^ at 0.5 mA cm^−2^/200 mF cm^−2^ at 20 mA cm^−2^	[190]
SCs	a/c‐CoS	Aqueous	1487.0 C g^−1^ at 1 A g^−1^/87.4% capacity retention after 5000 cycles at 1 A g^−1^	[203]
SCs	a/c‐Co(Al)S	Aqueous	1791.8 C g^−1^ at 1 A g^−1^/86.2% capacity retention after 5000 cycles at 1 A g^−1^	[188]
SCs	a/c‐NiCo‐sulfide/CC	Aqueous	250.16 mAh·g^−1^ at 1 A g^−1^/96.3% capacity retention after 1000 cycles at 7 A g^−1^	[204]
SCs	a‐Ni–Co–S/c‐MnS/rGO	Aqueous	1248 C g^−1^ at 2 A g^−1^/86.4% capacity retention after 5000 cycles at 10 A g^−1^	[205]
SCs	c‐Ni_9_S_8_@a‐Ni_2_B/CC	Aqueous	901.2 C g^−1^ at 1 A g^−1^/79.7% capacity retention over 5000 cycles at 10 A g^−1^	[81]
SCs	c‐NiMoO_4_@a‐Co–B	Aqueous	236.2 mAh g^−1^ at 1 A g^−1^/171.2 mAh g^−1^ at 20 A g^−1^	[44]
SCs	CC/c‐NiCo_2_O_4_@a‐NiCo‐P	Aqueous	1254.2 C g^−1^ at 1 A g ^−1^/1136 C g^−1^ at 16 A g ^−1^	[91]
SCs	a/c‐CoGa_2_O_4_‐S@c‐NiCo‐LDH	Aqueous	247.8 mAh g^−1^ at 1 A·g^−1^/143.1 mAh g^−1^ at 10 A g^−1^	[56]
SCs	a/c‐VO_2_	Nonaqueous	309 mAh g^−1^ at 0.1 A g^−1^ after 300 cycles/137.2 mAh g^−1^ after 3000 cycles at 1 A g^−1^	[206]
SCs	a‐VO*_x_* @c‐N‐MXene	Nonaqueous	222.2 mAh g^−^1 at 1 A g ^−1^/95.6% capacity retention after 1000 cycles at 1 A g^−1^	[194]

AC‐HNMs provide a new direction for further tuning the properties of nanomaterials, and open up an exciting research frontier. Despite some existing challenges in this field, given the unique structure and unprecedented properties of AC‐HNMs, we have reason to accelerate efforts to achieve greater progress in synthesis and applications, to better meet the needs of rapidly developing modern society.

## Conflict of Interest

The authors declare no conflict of interest.
